# In Silico Studies in Probing the Role of Kinetic and Structural Effects of Different Drugs for the Reactivation of Tabun-Inhibited AChE

**DOI:** 10.1371/journal.pone.0079591

**Published:** 2013-12-02

**Authors:** Rabindranath Lo, Nellore Bhanu Chandar, Manoj K. Kesharwani, Aastha Jain, Bishwajit Ganguly

**Affiliations:** 1 Computation and Simulation Unit (Analytical Discipline and Centralized Instrument Facility), CSIR-Central Salt & Marine Chemicals Research Institute, Bhavnagar, Gujarat, India; 2 Academy of Scientific and Innovative Research, CSIR-CSMCRI, Bhavnagar, Gujarat, India; Weizmann Institute of Science, Israel

## Abstract

We have examined the reactivation mechanism of the tabun-conjugated AChE with various drugs using density functional theory (DFT) and post-Hartree-Fock methods. The electronic environments and structural features of neutral oximes (deazapralidoxime and 3-hydroxy-2-pyridinealdoxime) and charged monopyridinium oxime (2-PAM) and bispyridinium oxime (Ortho-7) are different, hence their efficacy varies towards the reactivation process of tabun-conjugated AChE. The calculated potential energy surfaces suggest that a monopyridinium reactivator is less favorable for the reactivation of tabun-inhibited AChE compared to a bis-quaternary reactivator, which substantiates the experimental study. The rate determining barrier with neutral oximes was found to be ∼2.5 kcal/mol, which was ∼5.0 kcal/mol lower than charged oxime drugs such as Ortho-7. The structural analysis of the calculated geometries suggest that the charged oximes form strong O^…^H and N^…^H hydrogen bonding and C-H^…^π non-bonding interaction with the tabun-inhibited enzyme to stabilize the reactant complex compared to separated reactants, which influences the activation barrier. The ability of neutral drugs to cross the blood-brain barrier was also found to be superior to charged antidotes, which corroborates the available experimental observations. The calculated activation barriers support the superiority of neutral oximes for the activation of tabun-inhibited AChE compared to charged oximes. However, they lack effective interactions with their peripheral sites. Docking studies revealed that the poor binding affinity of simple neutral oxime drugs such as 3-hydroxy-2-pyridinealdoxime inside the active-site gorge of AChE was significantly augmented with the addition of neutral peripheral units compared to conventional charged peripheral sites. The newly designed oxime drug **2** appears to be an attractive candidate as efficient antidote to kinetically and structurally reactivate the tabun-inhibited enzyme.

## Introduction

Acetylcholinesterase (AChE, EC 3.1.1.7), one of the most important enzymes in many living organisms, is responsible for the catalytic hydrolysis of neurotransmitter acetylcholine during nerve signal transmission [1]–[3]. It is located at the neuromuscular junction and its catalytic triad (Ser203, Glu334 and His447 in *h*AChE) is mainly involved in the hydrolysis process [4], [5]. Many organophosphorus compounds (OPs) react irreversibly with acetylcholinesterase, inhibiting its catalytic activity and thus its control over the central nervous system [6]–[8]. Organophosphorus compounds (OPs) have been applied as pest control agents in agriculture and as chemical warfare agents in military conflicts and in terrorist attacks [9], [10]. The inhibition of AChE is mainly due to phosphylation (denoted by phosphorylation, phosphonylation, and phosphoamidation) of its active site serine hydroxyl group (Ser203 in *h*AChE), which is directly responsible for the catalytic hydrolysis of acetylcholine [11]. As a result of this inhibition, acetylcholine is not hydrolyzed, leading to overstimulation of cholinergic receptors which results in many medical disorders. The OP-AChE complex may further undergo aging process leading to dealkylation or deamination of the phosphorus conjugate [12]–[14]. The aging of enzymes is irreversible in nature, however, prior to aging the non-aged enzyme can be reactivated by strong nucleophiles like oximes.

Strategies for the development of effective drugs can be mainly classified into two categories: a kinetic approach [15]–[17] and a structural approach [18], [19]. Kinetic approaches involve the two step addition-elimination reaction process of drug molecules at the phosphorus center of OPs-inhibited AChE to form the phosphorylated drug and the reactivated enzyme. The efficiency of a reactivator drug is associated with its nucleophilicity and the activation barrier of that reaction process. The structural approach is related to the structure of the drug molecule and the AChE protein, which is also a challenge in the reactivation process. The most striking feature of the AChE structure is a deep and narrow gorge, ∼20 Å long, which perforates almost halfway along the enzyme. The active triad remains at the base of this gorge. For the drug to enter the active site gorge, the structural fluctuation of the enzyme plays an important role in opening the gorge every few picoseconds, leading to a fractal gating motion that allows passage to the substrate molecule (drug). Upon successful entry into the gorge, the substrate must move through the morphologically inhomogeneous pore surface to reach the active triad.

In earlier studies, research on the OP-AChE reactivation process mainly focused on the development of new and effective oximes. The high efficiency of oximes towards the reactivation process is mainly due to their high nucleophilicity and secondary interactions of their cationic moieties with the peripheral site of the enzyme [20]. The second cationic fragment of bis-quaternary oximes interacts with the peripheral site of the enzymes and gives extra stability to the drug inside the gorge, which makes them more effective compared to the monopyridinium oximes.

Recently, the blood-brain barrier (BBB) has been discussed as one of the challenges towards development of the drug for the reactivation process of OPs-inhibited AChE in the central nervous system (CNS). The blood-brain barrier is composed of an endothelial cell layer, which separates the circulating blood and the brain's extracellular fluid [21]. Nerve agents, being small lipophilic molecules, can easily cross the BBB and thereby inhibit AChE in the central nervous system (CNS). However, permanently charged cationic reactivators face difficulty in penetrating the BBB and thus, are less effective in reactivating AChE in the CNS. For example, HI-6 does not readily penetrate into the CNS and the mean BBB penetration ratio of 2-PAM was found to be approximately 10% [22].

Different approaches have been prompted for the development of oxime-based agents that can cross the BBB and reverse the effects of OP on AChE in the CNS. Recently, non-ionic reactivators have been synthesized, which can more easily penetrate the BBB and reactivate the inhibited *h*AChE [23]–[27]. Non-quaternary pyridinealdoxime compounds exhibited a high potency for the reactivation of VX and tabun-inhibited *h*AChE. The affinity towards the enzyme was suggested to be enhanced by linking the oxime with ligand through an alkyl or heteroalkyl chain fitting in *h*AChE gorge [24]–[27].

**Figure 1:**
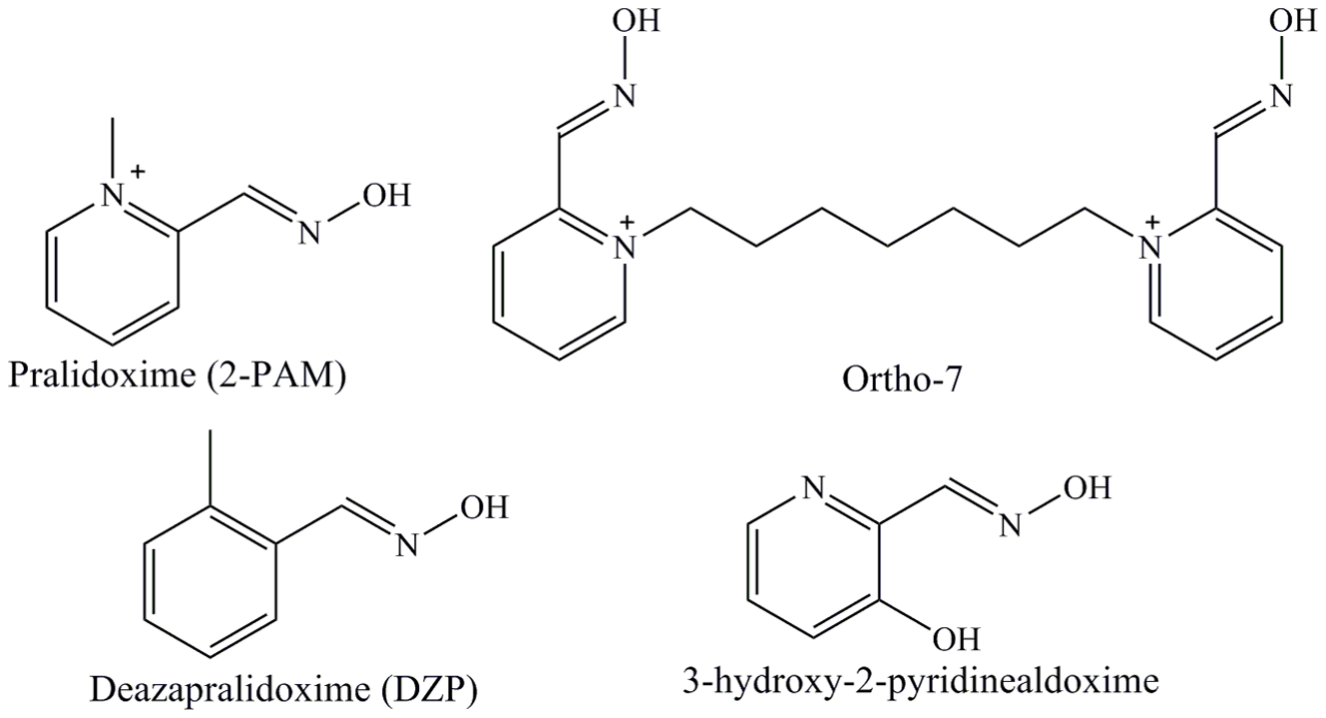
Chemical structures of oxime reactivators.

In this article, we have examined the efficiency of four drugs of different types towards the reactivation of the tabun-inhibited *m*AChE (Figure 1). Tabun is one of the oldest nerve agents, originally developed before World War II in Germany. Tabun has been chosen in this study due to the extraordinary resistant nature of tabun-AChE conjugates toward most of the oxime reactivators [28]. The steric hindrance in the tabun-inhibited AChE adduct and the weak electrophilicity of the phosphorus center makes this inhibited enzyme less reactive towards most of the oximes [29]. Here, we have examined the reactivation process of tabun-inhibited AChE with mono and bispyridinium oxime reactivators and neutral oximes in terms of both kinetics as well as from the structural point of view. The calculated potential energy surface shows that kinetically the neutral oximes are better reactivators compared to the charged antidotes. The docking study reveal that the peripheral site is also important in both enhancing the efficiency of such oxime drugs and their ability to be kinetically more active and to cross the BBB. These detailed analyses help to understand the factors responsible for the reactivation of tabun-inhibited AChE and assist in designing new types of drug for this reactivation process.

## Computational Methodology

Tabun-conjugated serine (SUN) residue with His447 imidazole ring was modeled from the reported PDB structure of tabun-conjugated mouse acetylcholinesterase (PDB code: 2JF0) [18]. This inhibited enzyme was optimized at M05-2X/6-31G* level in aqueous medium [30], [31]. All calculations were performed with Gaussian 09 suite program [32]. Full geometry optimization of all stationary points was performed in aqueous phase at the M05-2X/6-31G* energy level with polarizable continuum solvation model (PCM) using the integral equation formalism variant (IEF-PCM) [33]–[37]. The default UFF radii were used for the solvent calculations, which incorporates explicit hydrogen atoms. Single point calculations were performed at the MP2/6-31+G* level of theory to get accurate energies using M05-2X/6-31G* optimized geometries [38]–[39].

The stationary points were characterized by frequency calculations in order to verify that the transition structures had one, and only one, imaginary frequency. To verify that each saddle point connects two minima, intrinsic reaction coordinate (IRC) calculations of transition states were performed in both directions, that is, by following the eigenvectors associated to the unique negative eigen value of the Hessian matrix, using the González and Schlegel integration method [40]. The key equation for calculating rate constants from the Gibbs free energy in the present study is *k* = (k_B_T/*hc*°)e^−Δ*G°/RT^, where c° = 1 and the appropriate values are simply plugged into the other variables.

For the docking study, first we have taken the PDB of tabun-conjugated *m*AChE protein complexed with Ortho-7 (PDB code: 2JF0) from the Protein data bank. Hydrogen treatment and minimization of drug-protein complex was performed using Macromodel program [41]. The MMFF force field and the PRCG method was used to minimize the protein system. The minimized protein was further considered for the docking study using a grid based Autodock 4.2 program [42]. The simulation was performed with AutoDock, wherein the ligands explore six spatial degrees of freedom such as rotation and translation along with torsional degrees of freedom and the interaction energy was evaluated at each step to reach a global minimum. It employs a stochastic search algorithm called the Lamarckian Genetic Algorithm (LGA) to explore the grid space and hence, to perform energy evaluations of the position of the ligand with respect to the target energy grids. Autogrid was carried out for the preparation of the grid map using grid boxes of 70-70-70 Å and 100-100-100 Å, which also enclose the ligands. The genetic algorithm with local search (GALS) was used for the calculation of docking possibilities. The mode of interaction of Ortho-7 against 2JF0 was used as a standard docked model, the one used for calculation of root mean square deviation (RMSD) of docked inhibitors. Other drug molecules were optimized at M05-2X/6-31G* level of theory before further use in the docking study. Optimizations were performed using Gaussian 09 program [32]. It has been pointed out that although Autodock is apt for the docking simulations, calculated binding energy it produces are not always accurate [43]. Therefore, we have employed Macromodel using MMFF force field which supposedly gives more accurate binding energies compared to Autodock. The binding energy calculations were performed in Macromodel using the MMFF force field. The eq (1) was used to calculate the binding energy.
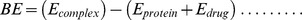
(1)


## Results and Discussion

All calculations have been performed for the reactivation process of tabun-inhibited *m*AChE with different oximes (Figure 1) at MP2/6-31+G(d)//M05-2X/6-31G(d) level of theory. The detailed mechanistic investigations for this reactivation process have been carried out in the aqueous medium using implicit polarizable continuum solvation model (PCM). Recently, molecular dynamics study has been performed with the antidote pralidoxime (2-PAM) and its corresponding neutral form deazapralidoxime (DZP) [44]. This study suggests that the positive charge of 2-PAM is important for its transportation to the active site of the enzyme. The kinetic pathways to reactivate the tabun-inhibited AChE with these drugs are not known. We have examined the potential energy surface for the reactivation of tabun-inhibited AChE with pralidoxime (2-PAM) and its corresponding neutral form deazapralidoxime (DZP) (Figure 1). Further, the study has been extended to bisquaternary pyridinium Ortho-7 and neutral oxime 3-hydroxy-2-pyridinealdoxime (Figure 1). The detailed mechanistic study for the reactivation process of tabun-inhibited AChE with charged oximes 2-PAM, Ortho-7 and neutral drugs DZP and 3-hydroxy-2-pyridinealdoxime help to segregate the role of positive charge on oxime drugs towards the reactivation kinetics.

**Figure 2:**
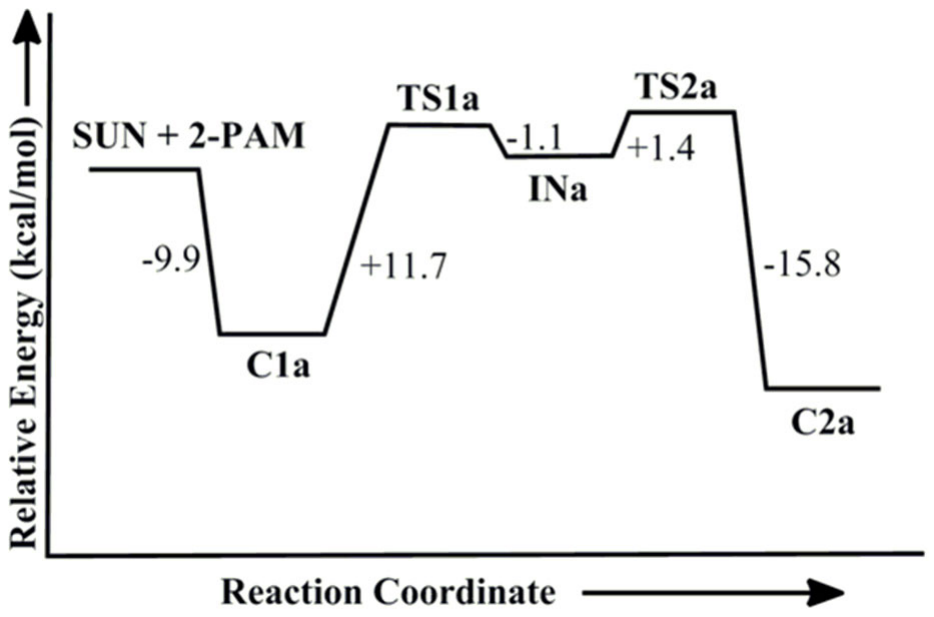
MP2/6-31+G*//M05-2X/6-31G* calculated energy profile diagram for the reactivation of tabun-inhibited AChE with 2-PAM in aqueous phase.

**Figure 3:**
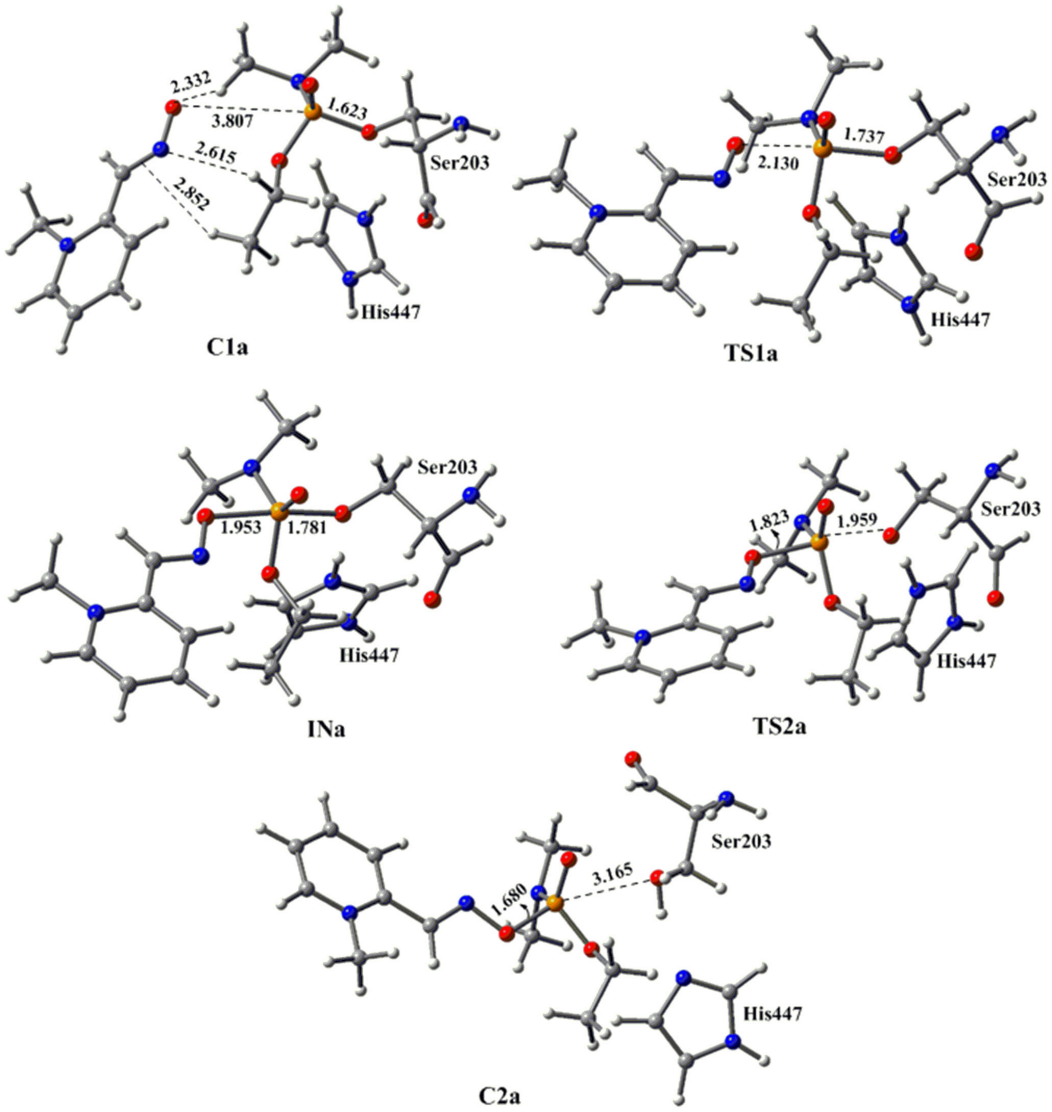
M05-2X/6-31G* optimized geometries and selected bond distances (Å) for species involved in the reactivation process of tabun-conjugated serine (SUN) molecule with pralidoxime (2-PAM) in aqueous phase. (red = oxygen; blue = nitrogen; white = hydrogen; yellow = phosphorus; gray = carbon).

The optimized geometry of tabun-conjugated serine (SUN) moiety with imidazole ring derived from tabun-inhibited acetylcholinesterase was taken for the reactivation process with pralidoxime (2-PAM). The MP2/6-31+G*//M05-2X/6-31G* calculated potential energy surface and M05-2X/6-31G* optimized geometries for this reactivation process with 2-PAM are given in Figures 2 and 3, respectively. The oxime nucleophile 2-PAM attacks the phosphorus atom of nerve agent from the opposite side of serine residue (Figure 3). The reactivation process of tabun-inhibited AChE adducts with 2-PAM proceeds via an addition-elimination pathway with involvement of a trigonal bipyramidal intermediate (Figure 3) [45]–[47]. After complexation with tabun-inhibited enzyme the nucleophile 2-PAM becomes energetically more stable by 9.9 kcal/mol compared to the separated reactants (Figure 2). The attack of 2-PAM to the P center of the inhibited enzyme occurs through a transition state **TS1a**, which is 11.7 kcal/mol higher in energy compared to the stable complex **C1a** (Figure 2). The calculated potential energy surface shows the rate determining barrier of 12.0 kcal/mol for the reactivation process of 2-PAM and is governed by the second transition state (**TS2a**) i.e. the breaking of P-O_(ser)_ bond (Figures 2 and 3).

**Figure 4:**
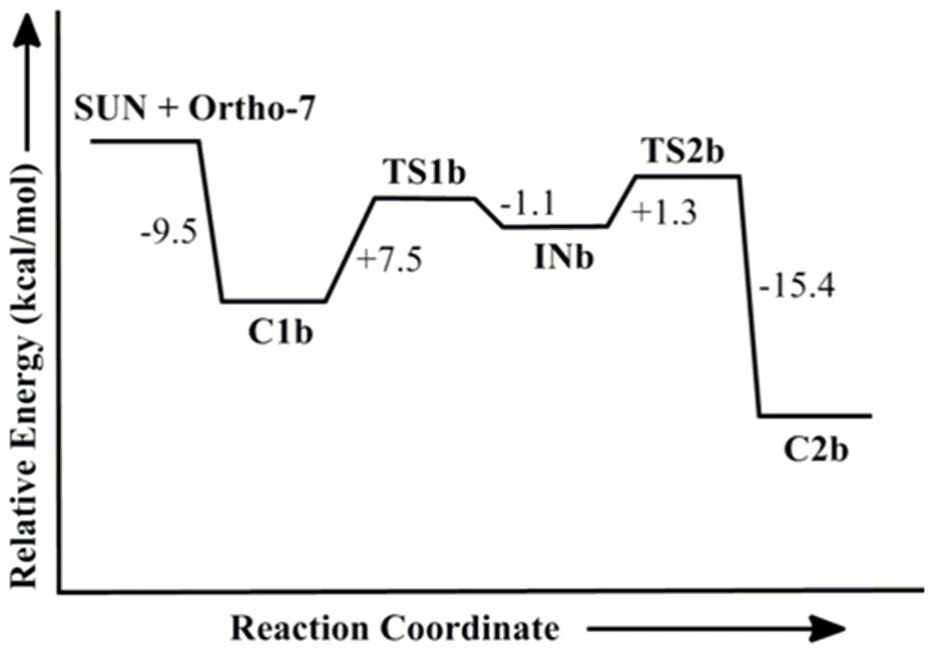
MP2/6-31+G*//M05-2X/6-31G* calculated energy profile diagram for the reactivation of tabun-inhibited AChE with Ortho-7 in aqueous phase.

**Figure 5:**
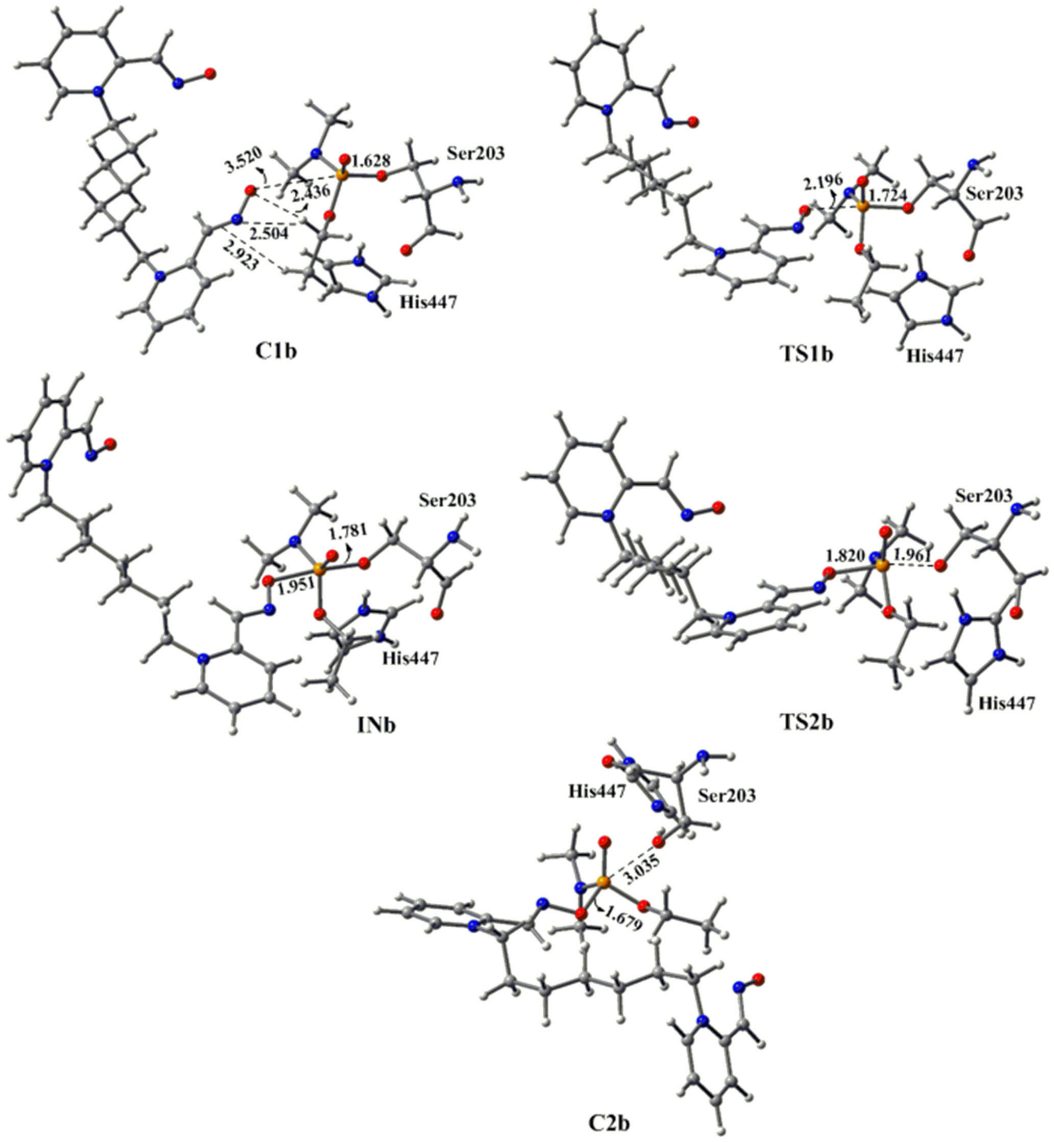
M05-2X/6-31G* optimized geometries and selected bond distances (Å) for species involved in the reactivation process of tabun-conjugated serine (SUN) molecule with Ortho-7 in aqueous phase. (red = oxygen; blue = nitrogen; white = hydrogen; yellow = phosphorus; gray = carbon).

Further, we have extended our study by considering bispyridinium oxime for the reactivation of tabun-inhibited AChE as monopyridinium oximes are not able to sufficiently reactivate this inhibited enzyme. The oxime potency can be improved by connecting two pyridinium rings with a central linker. It has been reported that Ortho-7 is a more potent drug for the reactivation of tabun inhibited AChE [18]. The presence of an aromatic group in the side chain improves the interactions with AChE residues via π-π and cation-π interactions. The length of the linker between the two pyridinium rings also has a significant role in enhancing the lipophilicity of the drug [20]. The reactivation process of tabun-inhibited enzyme with Ortho-7 has been examined at the same level of theory. The potential energy diagram shows that the reactivation process with Ortho-7 also follows the addition-elimination pathway with the involvement of a trigonal bipyramidal intermediate as observed in the case of 2-PAM. The MP2/6-31+G*//M05-2X/6-31G* calculated energy surface diagrams and M05-2X/6-31G* optimized geometries are given in Figures 4 and 5, respectively. Ortho-7 also showed similar potential energy surface to that obtained with 2-PAM (Figures 2 and 4). However, the calculated PES suggests that the initial attack of the oxime to the phosphorus center is favoured for Ortho-7 compared to 2-PAM. The CHelpG charge analysis shows that the larger negative charge resides on the oxygen atom of Ortho-7 (−0.5063) compared to 2-PAM (−0.4371) (Table 1). This charge analysis indicates that Ortho-7 will be a better nucleophile compared to 2-PAM [48]. The Wiberg bond index calculated for the P^…^O_(Ortho-7)_ bond of **C1b** is found to be 0.008 au, which is higher than that of P^…^O_(2-PAM)_ bond (0.005 au) for **C1a**, implying a stronger interaction in the reactant complex of Ortho-7 compared to 2-PAM [45], [46]. The calculated bonding parameters also suggest the preferable interaction of Ortho-7 with tabun-inhibited AChE (Figures 3 and 5). The rate determining step for the reactivation of tabun-inhibited AChE with bispyridinium oxime Ortho-7 is also governed by the second transition step with an energy barrier of 7.7 kcal/mol, which is 4.3 kcal/mol lower than the corresponding barrier with 2-PAM. These calculated energy profiles correlate well with the experimental observation that the monopyridinium oxime (2-PAM) is less reactive than the bispyridinium oxime (Ortho-7) for the reactivation of tabun inhibited *m*AChE [20].

**Table 1:** CHelpG charges (in units of electrons) at the oxygen atom of the oximes in aqueous phase.

oximes	CHelpG
2-PAM	−0.4371
Ortho-7	−0.5063
DZP	−0.5949
3-hydroxy-2-pyridinealdoxime	−0.5329

Mono and bispyridinium oxime reactivators have been used in the reactivation process of tabun-inhibited AChE; however, these drugs possess several drawbacks. One of the major hurdles is to cross the BBB and reactivate AChE in the central nervous system [20]. This arises due to the presence of permanent charge on these antidotes. Therefore, alternative antidotes are required, which can pass the BBB more efficiently to reactivate AChE in the central nervous system. Hence, the choice could be a neutral antidote to overcome this situation.

**Figure 6:**
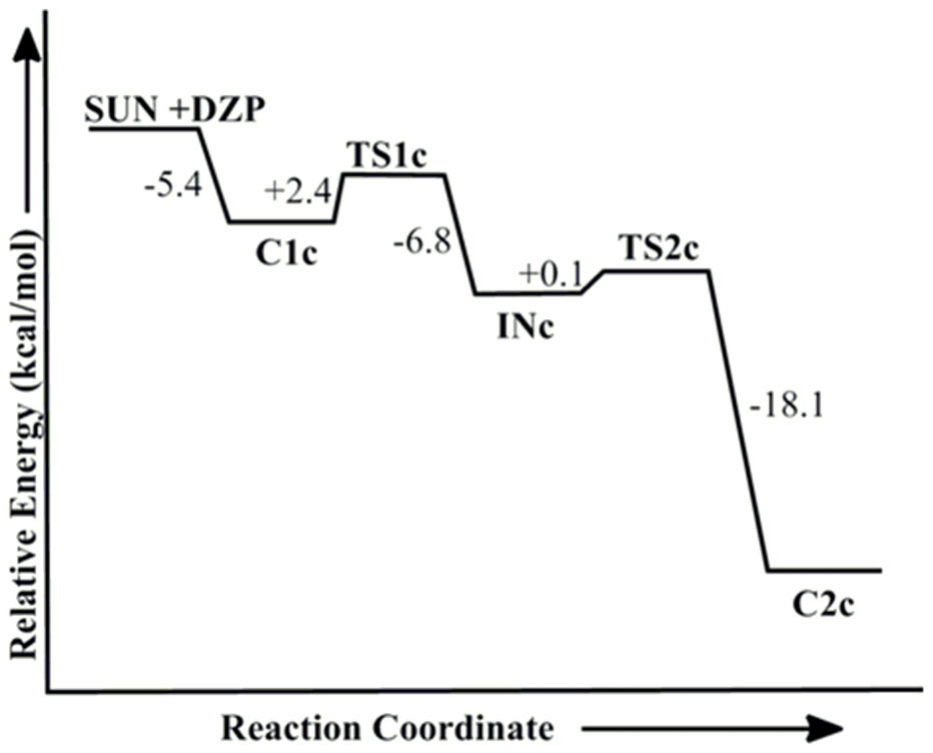
MP2/6-31+G*//M05-2X/6-31G* calculated energy profile diagram for the reactivation of tabun-inhibited AChE with deazapralidoxime (DZP) in aqueous phase.

**Figure 7:**
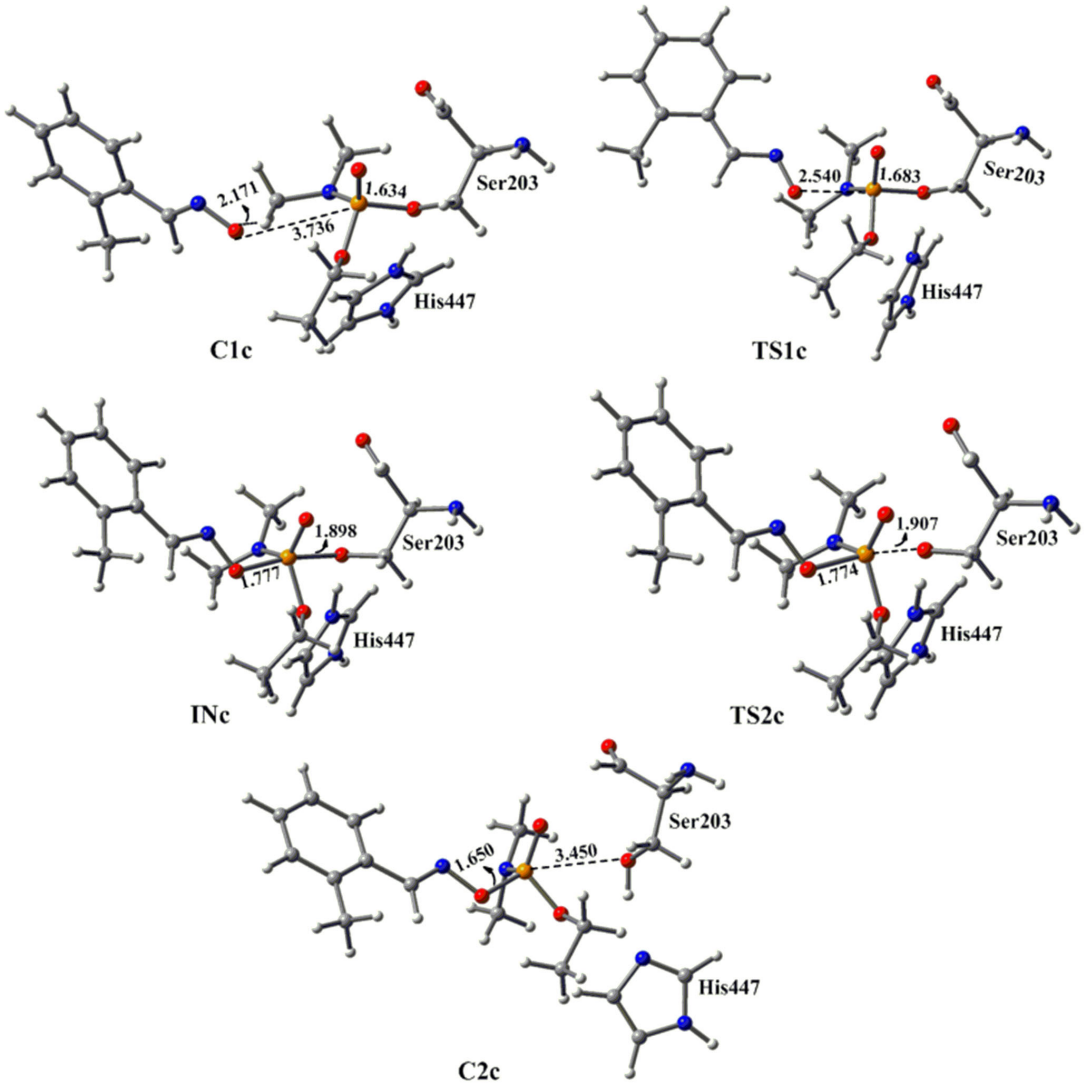
M05-2X/6-31G* optimized geometries and selected bond distances (Å) for species involved in the reactivation process of tabun-conjugated serine (SUN) molecule with deazapralidoxime (DZP) in aqueous phase. (red = oxygen; blue = nitrogen; white = hydrogen; yellow = phosphorus; gray = carbon).

We have examined the potential energy surface for the reactivation of tabun-inhibited AChE with the neutral form of 2-PAM i.e., deazapralidoxime (DZP). The optimized geometry of tabun-conjugated serine (SUN) moiety with the imidazole ring derived from tabun-inhibited acetylcholinesterase, was taken for the reactivation process with deazapralidoxime (DZP). The MP2/6-31+G*//M05-2X/6-31G* calculated potential energy surface and M05-2X/6-31G* optimized geometries for the reactivation process with deazapralidoxime (DZP) are given in Figures 6 and 7, respectively. The reactivation process of tabun-inhibited AChE adducts with DZP also proceeds via an addition-elimination pathway with the involvement of a trigonal bipyramidal intermediate (Figure 7). DZP initially forms a complex with the substrate (**C1c**) which is 5.4 kcal/mol lower in energy than the separated reactants (Figures 6 and 7). This complex further proceeds to the transition state (**TS1c**), in which DZP molecule attacks on the phosphorus center of the tabun inhibited serine molecule. The MP2/6-31+G* calculated activation barrier for the transition state **TS1c** has been found to be 2.4 kcal/mol with respect to the complex **C1c** which is much lower compared to the charged oxime 2-PAM (Figures 2 and 6). The DZP attacks opposite to the serine moiety in nearly linear (∠O-P-O = 172.6°). In **TS1c** the P-O_(DZP)_ and P-O_(ser)_ distances are 2.540 Å and 1.683 Å, respectively. The Wiberg bond index calculated for the P^…^O_(DZP)_ bond of **TS1c** has been found to be 0.11 au, which is 0.10 au higher than that of **C1c**, implying a stronger interaction in the transition state **TS1c** compared to the reactant complex **C1c**. Transition state **TS1c** further proceeds to a trigonal bipyramidal intermediate **INc**, which is 6.8 kcal/mol lower in energy than **TS1c**. In intermediate **INc** the P-O_(DZP)_ and P-O_(ser)_ distances change to 1.777 Å and 1.898 Å, respectively. The calculated Wiberg bond index of 0.48 au for P^…^O_(DZP)_ bond also supports for the strengthening of the bond between the incoming nucleophiles and the phosphorus center. The trigonal bipyramidal intermediate (**INc**) further proceeds to the product complex **C2c** via a second transition state **TS2c** with very small activation barrier (Figure 6). In the transition state **TS2c**, the smaller value of the calculated Wiberg bond index for P-O_serine_ bond (0.35 au) suggests breaking of tabun-serine interaction. During the whole reactivation process the proton of His447 interacts with the oxygen of serine residue (Figure 7). The reactivation process between the deazapralidoxime and tabun-conjugated serine is governed by the first step, i.e. the attack of nucleophile in the phosphorus center of the tabun inhibited enzyme (Figures 6 and 7). The following steps are downhill in nature at MP2/6-31+G*//M05-2X/6-31G* level of theory. The calculated energy profile suggests that deazapralidoxime reactivates the tabun-inhibited AChE with a very small energy of activation compared to 2-PAM (Figures 2 and 6).

These calculated results reveal that the charge bearing antidote (2-PAM and Ortho-7) would be less effective in reactivating the tabun-inhibited AChE compared to the neutral antidotes. The better efficiency of the neutral antidotes in terms of the kinetic point of view seems to arise due to higher nucleophilicity than the charged species. The CHelpG charge analysis shows that the nucleophilic oxygen (-N-O^−^) of DZP carries much greater negative charge compared to 2-PAM and Ortho-7 (Table 1), indicating a stronger interaction in the reactant complex of neutral oxime with the phosphorus center compared to the charged one. Further, it appears that geometrical parameters can also contribute to influence the activation barriers. The structural analysis shows that 2-PAM and Ortho-7 forms strong O^…^H and N^….^H hydrogen bonding and C-H^…^π non-bonding interaction with the tabun-inhibited enzyme complex (**C1a** and **C1b**, respectively), whereas the neutral oxime DZP forms only O^…^H hydrogen bonding (**C1c**) (Figures 3, 5 and 7). Such a difference in interactions contributes to an increased stabilization of the complex with charged oximes relative to the neutral ones. The PES shows that the first transition state **TS1c** for DZP more resembles the complex (**C1c**), and can be considered as an early transition state [49], [50]. On the other hand, the transition state (**TS1a**) involving 2-PAM is considered to be the late transition state due to its close resemblance with the intermediate (**INa**). This is related to the barrier heights— the early to late transition states enhances the activation barrier [49], [50].

We have also examined the reactivation process with another neutral form of 2-PAM i.e., 2-pyridinealdoxime. The calculated PES suggests that the reactivation process also followed the same pathway as observed with DZP (Figures S1 and S2). The first step of the reaction is the rate determining step of the process with the activation barrier of 2.4 kcal/mol (Figure S1), which is much lower compared to monoquaternery oxime 2-PAM (Figure 2). This result suggests that 2-pyridinealdoxime can also reactivate the tabun-inhibited AChE more efficiently than its charged analogue 2-PAM.

**Figure 8:**
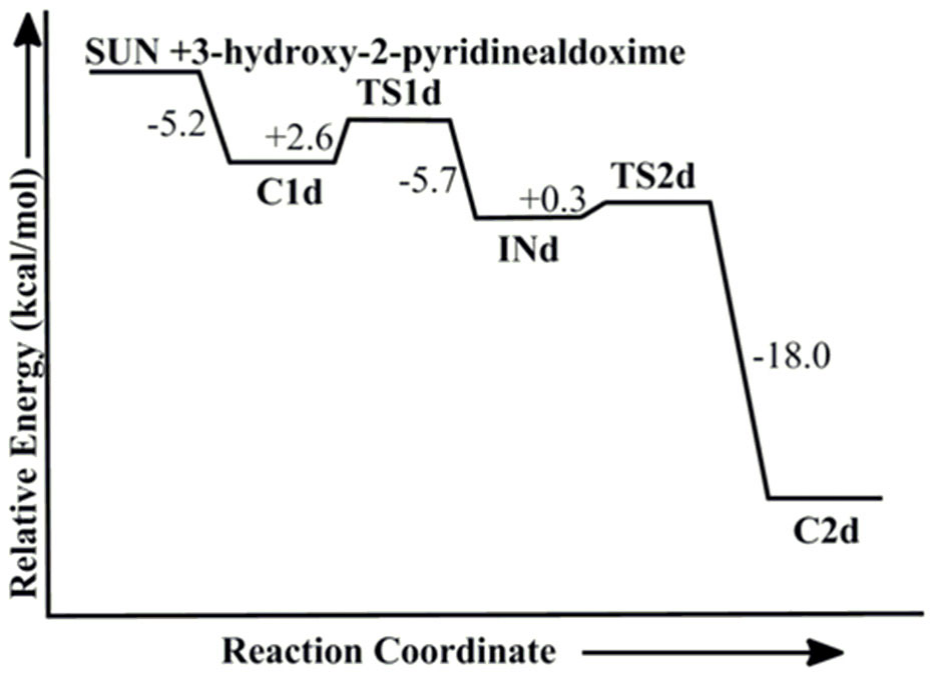
MP2/6-31+G*//M05-2X/6-31G* calculated energy profile diagram for the reactivation of tabun-inhibited AChE with 3-hydroxy-2-pyridinealdoxime in aqueous phase.

**Figure 9:**
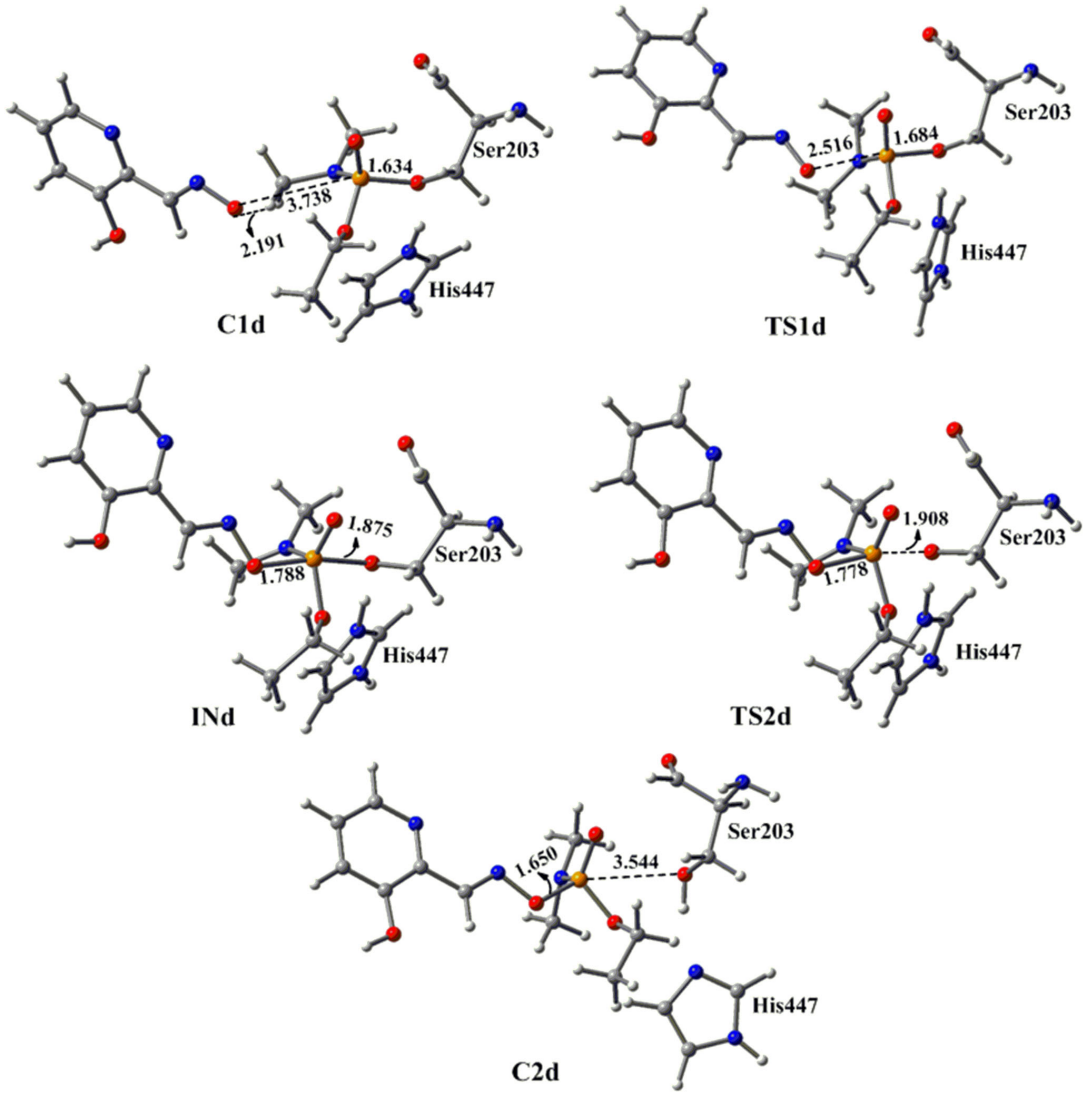
M05-2X/6-31G* optimized geometries and selected bond distances (Å) for species involved in the reactivation process of tabun-conjugated serine (SUN) molecule with 3-hydroxy-2-pyridinealdoxime in aqueous phase. (red = oxygen; blue = nitrogen; white = hydrogen; yellow = phosphorus; gray = carbon).

Recently, the ability of neutral reactivator pyridinealdoxime to reactivate OP inhibited enzyme was reported [23]–[27]. This pyridinealdoxime showed a considerable capacity to reactivate VX-, tabun- and ethyl paraoxan inhibited human AChE [27]. These neutral drugs possess the capability to diffuse across the BBB and reactivate the AChEs in the central nervous system. Further, such β-hydroxy oximes prevent the recapture phenomenon by undergoing intramolecular cyclization with the organophosphorus ester [25]. The attack of hydroxyl group onto the nitrogen centre rapidly forms the isoxazole ring, which retards the inhibition process of catalytically active serine with activated phosphorylamidoxime. Here, we have examined the efficiency of 3-hydroxy-2-pyridinealdoxime towards the reactivation of tabun-inhibited acetylcholinesterase at the same level of theory. It has been observed that the non-quaternary oxime 3-hydroxy-2-pyridinealdoxime effectively reactivates VX-poisoned human AChE with a reactivation rate constant of *k*_r_ = 0.5±0.1 min^−1^, pH 7.0, 25°C [24]. However, this oxime has a very large dissociation constant *K*_D_ = 32±11 mM, which implies that it has a weak affinity towards the inhibited AChE [24]. The calculated PES shows that the reactivation process with 3-hydroxy-2-pyridinealdoxime also follows the addition-elimination pathway involving a trigonal bipyramidal intermediate. The MP2/6-31+G*//M05-2X/6-31G* calculated energy surface diagrams and optimized geometries are given in Figures 8 and 9, respectively. The PES showed that this pyridinealdoxime also follows similar potential energy surface as obtained with neutral system DZP (Figures 6 and 8). The calculated results suggest that the first step in this process is the rate determining step i.e. the attack of pyridinealdoxime to the phosphorus center of tabun-inhibited enzyme (Figure 8). The computed results show that the activation barrier for the reactivation process of pyridinealdoxime is 2.6 kcal/mol, which is comparable to the activation barrier calculated for DZP (Figures 6 and 8). The lower activation barrier in the case of pyridinealdoxime also suggests the importance of nucleophilicity, geometrical parameters and the formation of early transition state (Table 1, Figures 6 and 8).

The potential energy surface calculated at MP2/6-31+G*//M05-2X/6-31G* level shows that kinetically the neutral oximes are better reactivators compared to the charged antidotes. We have further calculated the rate constants from the Gibbs free energies for the rate-determining steps of the reactivation processes. The first order rate constants for the reactivation reactions involving the neutral oximes DZP and 3-hydroxy-2-pyridinealdoxime are 1.9×10^10^ s^−1^ and 5.1×10^9^ s^−1^, respectively [45], [46]. In the case of charged oximes, the rate constants values are relatively lower 4.0×10^7^ s^−1^ and 4.1×10^3^ s^−1^ for Ortho-7 and 2-PAM, respectively. Experimental studies revealed that the reactivation rate constants for the VX-*h*AChE were found to be higher for 3-hydroxy-2-pyridinealdoxime compared to 2-PAM, which is in good agreement with the calculated results [26]. However, the overall second-order rate constant for reactivation is favoured for the charged 2-PAM compared to the neutral 3-hydroxy-2-pyridinealdoxime [26].

Commonly used reactivators having permanently charged cationic compounds have less tendency to cross the BBB [20]. For 2-PAM, the BBB penetration (striatal extracellular/blood concentration ratio) was estimated to be approximately only 10% using the *in vivo* rat brain microdialysis technique [20]. The diffusion of oximes into the BBB depends upon their lipid solubility and is inversely proportional to their degree of ionization [51]. To examine the lipophilicity of the drugs and their penetration to the blood-brain barrier, LogP values were calculated [21], [52]. The LogP values are determined as the ratio of concentrations of a particular compound in the two phases of a mixture of two immiscible solvents at equilibrium. Hence, these coefficients are a measure of differential solubility of the compound between the two solvents. The octanol-water partition coefficient (LogP) has first been shown to yield correlation with biological activities by Hansch and Fujita *et al* [53]. LogP value indicates the measure of lipophilicity/hydrophilicity of the compounds. Lipophilicity plays an important role in rational drug design as it is of primary significance in drug absorption and distribution. The octanol/water partition coefficient (LogP) calculation was performed using the PrologP module of the Pallas 3413 software [54]. Oximes are in general polar compounds, particularly when they are charged, and hence they are highly soluble in water. A negative value of LogP reflects the hydrophilic nature of the oximes and thus such oximes have a lower tendency to penetrate the BBB [51]. Various permanent charged bis-quaternary oximes such as HI-6, obidoxime (logP<−3) and BI-6, K-27 and K-48 (logP<−2.5) show a greater hydrophilic nature and thereby show lower penetration across the minimal blood-brain-barrier (BBB) [51]. In the case of Ortho-7, the Log P value was found to be −1.98 indicating its poor penetration to the blood-brain barrier (Table 2). 2-PAM shows a highly negative LogP value (−2.38) in the series and thereby shows lower diffusion inside the blood-brain barrier (Table 2). The lipophilicity is increased in the case of uncharged drugs, which suggests enhanced BBB permeability. The neutral drugs DZP and 3-hydroxy-2-pyridinealdoxime shows positive LogP value, indicating better penetration to the blood-brain barrier compared to the charged oximes. The LogP value for DZP was found to be 1.95, which is highest in the series (Table 2). The calculated LogP values suggest that the neutral oximes are less soluble in water. These results corroborate the higher tendency for the neutral antidotes to cross the BBB [20].

**Table 2:** The octanol–water partition coefficient (LogP) of different oximes.

Oxime	LogP
Ortho7	−1.98
2-PAM	−2.38
DZP	1.95
3-hydroxy-2-pyridinealdoxime	0.43
**1**	4.14
**2**	5.60

From the above results, it can be hypothesized that neutral oximes might be better drugs for the reactivation of tabun-inhibited AChE in terms of the kinetic approach and the diffusion through BBB. However, it is well reported that the structural approach, i.e. the interaction of drug with enzyme residues plays an important role towards the reactivation process [18], [19]. To examine the role of peripheral interactions between the neutral drug and the enzyme, further calculations have been performed. These calculated results have been compared with the analogous study of charged oximes. We have examined the interaction energy of studied charged and neutral oximes with whole AChE protein by using docking studies in Autodock followed by binding energy calculations using MMFF force field to obtain more reliable energies. Molecular docking programs have been useful to understand the binding mode of a ligand in the active sites of a protein [55]. Such studies have been found to be useful in predicting the binding affinities for human AChE inhibitors [56]. We have generated a series of charged and neutral oximes bound AChE structures based on the affinity-based rank order.

**Figure 10:**
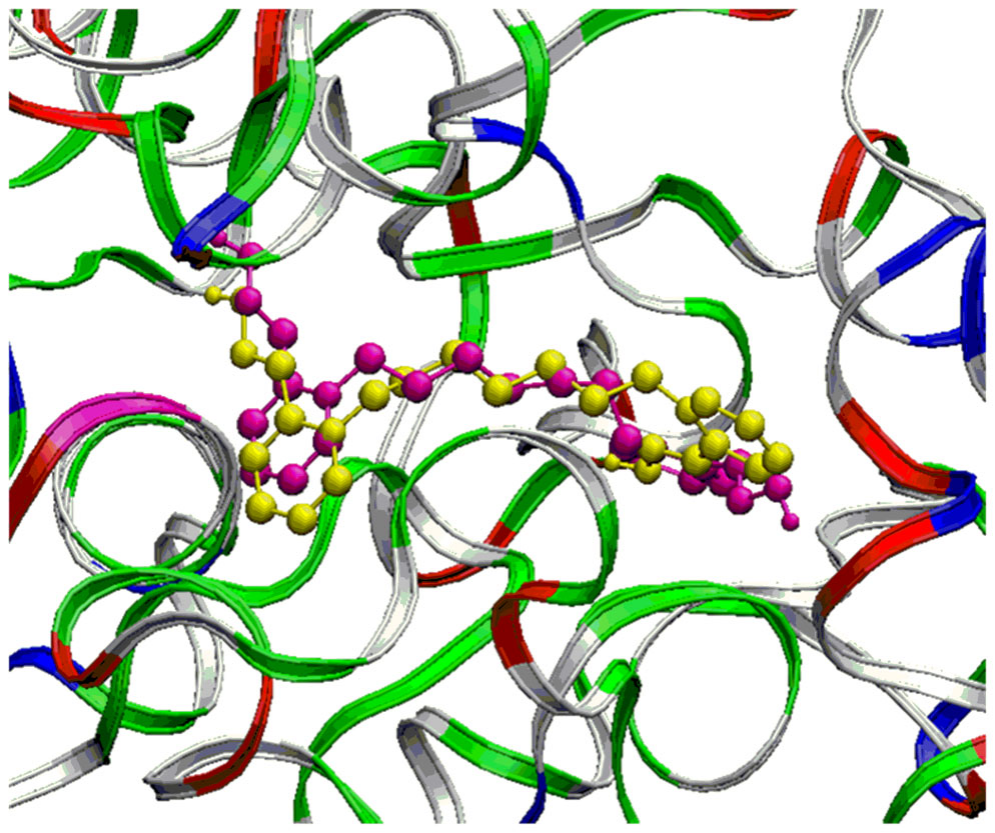
The overlapping between the crystal structure of tabun-inhibited AChE with Ortho-7 and the docked conformation of Ortho-7 with tabun-inhibited AChE. (Yellow = crystal structure of tabun-inhibited AChE with Ortho-7 and Magenta = docked conformation of tabun-inhibited AChE with Ortho-7).

The crystal structure of tabun-inhibited *m*AChE with drug Ortho-7 is available in literature, which enables us to examine this reactivation process in real system [18]. The quality and correctness of the docking results can be deduced from the calculation of root-mean-square deviation (RMSD) [55]. We have carried out the docking study with positively charged bis-quaternary pyridinium oxime Ortho-7 with tabun-inhibited AChE. To compare the performance of docking study with the available single crystal X-ray structures, we have performed an overlapping between the crystal structure of tabun-inhibited AChE with Ortho-7 and the docked conformation of Ortho-7 with tabun-inhibited AChE. The overlapped results are shown in the Figure 10. The results show that the docked conformation shows good correlation of RMSD value 2.73 and close overlap with the crystal structure of tabun-inhibited AChE with Ortho-7. The oxime oxygen of Ortho-7 is at a distance of 5.37 Å from the phosphorus of tabun molecule, which is close to the distance reported in the crystal structure (6.74 Å) [18]. Ortho-7 was mainly associated with the aromatic residues via cation-π and π-π interactions. One pyridinium ring is sandwiched between the aromatic residues viz. Tyr72 and Trp286 via a cation-π interaction at the entrance of the active site gorge (Figure S3). The second pyridinium site interacts with the phenyl ring of Tyr337 in the vicinity of the choline binding site and a T-shaped interaction occurs with the indole ring of Trp86 (Figure S3). These structural analyses show a good correlation with the crystal structure [18]. The calculated binding energy for the Ortho-7 complex is −38.1 kcal/mol with MMFF force field (Table 3). Now, we have extended our study with the positively charged monoquaternary pyridinium oxime 2-PAM and the neutral drugs DZP and 3-hydroxy-2-pyridinealdoxime with tabun-inhibited *m*AChE. The binding energy calculated for 2-PAM is −27.6 kcal/mol, which is lower than bis-cationic oxime Ortho-7 (Table 3). The docking geometry of 2-PAM shows that the pyridinium ring interacts with the aromatic residue of Tyr72 (Figure S4Two neutral drugs DZP and 3-hydroxy-2-pyridinealdoxime show lower binding energy compared to the cationic drugs Ortho-7 and 2-PAM (Table 3). The docking geometry show that DZP interacts with Phe297 and Phe338 via π-π interaction (Figure S5). In the case of 3-hydroxy-2-pyridinealdoxime, the calculated binding energy (−10.5 kcal/mol) is the lowest in the series (Table 3). There is a weak interaction between the neutral drug molecule with the amino acid residue of the enzyme. 3-hydroxy-2-pyridinealdoxime interacts with Arg247 and Arg296 via cation-π interaction between the cationic side of Arg residues with the aromatic ring of the oxime (Figure S6). Amongst all the docking geometries, the neutral molecules show less interaction with the inhibited protein molecule compared to the charged oximes. The bisquaternary pyridinium oxime Ortho-7 shows the highest binding energy of −38.1 kcal/mol in the series (Table 3). Further, we have examined the same docking study with a grid box of 100 Å (x, y and z). The enhancement of dimensions of the grid box enables the oximes to move freely and rotate in the docking space. The calculated binding energies also follow the same trend as we observed in the previous docking study with a grid box of 70 Å (x, y and z) (Table S1).

**Table 3:** Binding energies (kcal/mol) calculated with MMFF force field of drug-tabun-conjugated-AChE complexes.

	Binding Energy
Ortho7	−38.1
2-PAM	−27.6
DZP	−22.8
3-hydroxy-2-pyridinealdoxime **1 2**	−10.5 −30.9 −31.6

We have also extended the docking study with 45 crystal water molecules in mouse AChE to consider the effect of explicit solvent molecules. The docking results show that the calculated binding energies in the MMFF force field, are greatly enhanced in presence of water molecules for charged oximes (Table S1).

The docking study revealed that the peripheral site is also important in enhancing the efficiency of such oxime drugs besides their ability to be kinetically more active and to cross the BBB. The reactivation ability of the bisquaternary compound Ortho-7 also depends on the interaction of second pyridinium ring with the peripheral anionic site of AChE [18], [19]. However, the presence of such charged rings precludes them from entering the blood-brain barrier (BBB). Therefore, the design of a better antidote for the reactivation of tabun-conjugated AChE requires a control on both the kinetic and the structural aspects to penetrate the active site of the gorge of AChE as well as the BBB.

**Figure 11:**
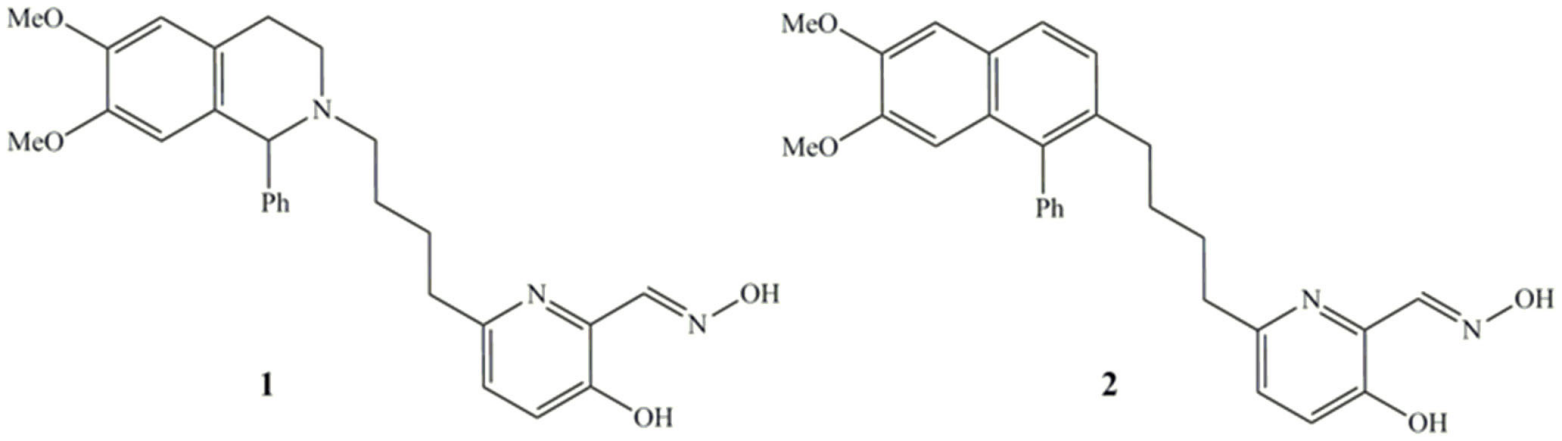
Chemical structures of neutral oxime reactivators.

**Figure 12:**
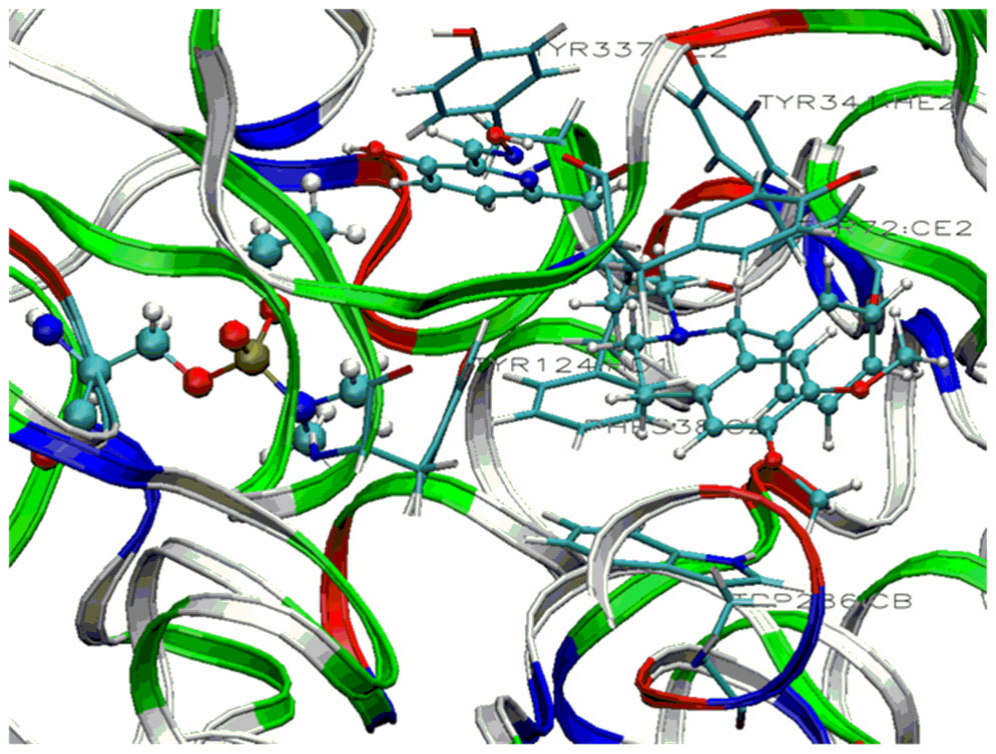
Stable conformation of tabun-inhibited AChE with oxime 1 showing interaction with residues of peripheral anionic site and active site. (Cyan = carbon, blue = nitrogen, white = hydrogen and red = oxygen) (Residues of peripheral anionic site are shown in tube format).

**Figure 13:**
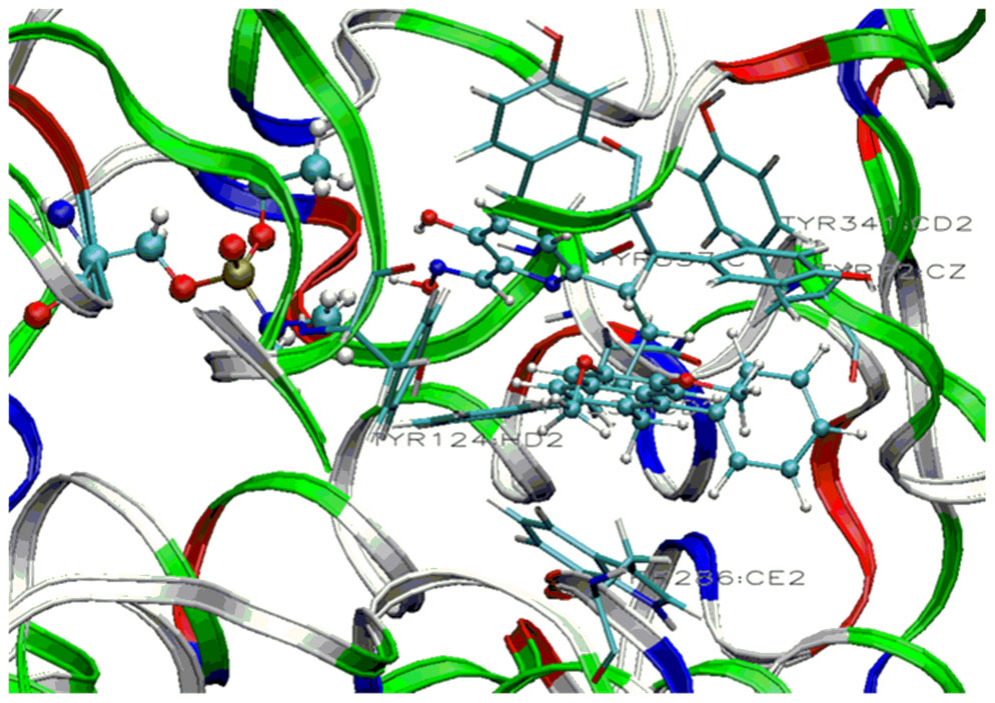
Stable conformation of tabun-inhibited AChE with oxime 2 showing interaction with residues of peripheral anionic site and active site. (Cyan = carbon, blue = nitrogen, white = hydrogen and red = oxygen) (Residues of peripheral anionic site are shown in tube format).

The docking studies revealed a different picture, where the charged drug (Ortho-7) is a better candidate to reach the active-site in AChE through non-covalent interactions with the peripheral sites of the enzyme. This study prompted us to tune the affinity of uncharged oxime 3-hydroxy-2-pyridinealdoxime with a neutral side chain (Figure 11). Studies in which the affinity of a neutral drug has been enhanced by appropriate structural modifications are limited in the literature [24], [27]. Nonquaternary phenyltetrahydroisoquinoline pyridinealdoxime conjugates were prepared to achieve better drug efficacy than pyridinium oximes (2PAM, Obidoxime, Trimedoxime) in reactivating VX-, tabun- and ethyl paraoxan inhibited human AChE [27]. The docking studies performed with the side chain attached to the reported drug **1** showed that the binding energy is enhanced significantly compared to the drug 3-hydroxy-2-pyridinealdoxime (Table 3). Oxime **1** is 1-2 order of magnitude more efficient in reactivating VX- and tabun-inhibited AChE than 2-PAM [27]. The peripheral ligand of oxime **1** interacts with the aromatic residues of Trp286 and Tyr72 via π-π interaction at the entrance of the active site gorge (Figure 12). The neutral pyridinium ring interacts with the phenyl ring of Tyr337 in the vicinity of the choline binding site. In addition to that, some C-H…π interactions with the residues viz. Tyr124, Phe338, Tyr341 occur (Figure 12). We have further extended this study with a naphthyl group attached to the drug **2** of similar chain length (Figure 11). A chain length of four and five carbon atoms attached on position 6 of the pyridine ring, is suitable for good reactivation efficiency [27]. The naphthalene system can induce better π-π interactions with the peripheral sites of AChE and hence can enhance the binding affinity inside the enzyme. The docking results support this hypothesis and the binding energy was found to be −31.6 kcal/mol. The binding energy calculated at MMFF force field shows that there is a significant increase in the binding energy in the designed drug (**2**) compared to the other neutral antidotes (Table 3). The oxime **2** also involves non-covalent interactions with the aromatic residues in the inhibited AChE. The naphthalene moiety interacts with the aromatic residues viz. Trp286 and Tyr 337 via π-π interaction at the entrance of the active site gorge (Figure 13). On the other hand, the aromatic residues Tyr72, Tyr124, Phe338, Tyr341 involves C-H…π interaction with naphthalene ring of the oxime **2** (Figure 13). Importantly, the newly designed drug (**2**) shows much higher LogP value (5.60) than the other neutral oximes (Table 2). The higher LogP value of **2** indicates its poor solubility in water and greater lipophilicity of the oximes and suggests a greater capability for crossing the BBB. These calculated results show that the efficiency of an antidote can be significantly improved by selecting the appropriate active site and the ligand attached to it to overcome the possible hurdles faced by the existing drugs.

## Conclusions

In this work, we have examined the reactivation mechanisms of tabun-conjugated AChE with neutral oximes (deazapralidoxime and 3-hydroxy-2-pyridinealdoxime) and the charged oximes (2-PAM, Ortho-7) using DFT and post-Hartree-Fock calculations. This study presents comprehensive analyses of kinetic and structural approach of neutral and charged drugs towards the reactivation of tabun-inhibited AChE. With all studied charged and neutral oximes, the reactivation process proceeds with addition-elimination process. For the reactivation process with neutral oximes (DZP and 3-hydroxy-2-pyridinealdoxime), the initial step is the rate determining step, whereas, the transition state located with the elimination of serine group is the rate determining step for the charged oximes (2-PAM and Ortho-7). The non-covalent interactions such as O^…^H and N^…^H hydrogen bonding and C-H^…^π non-bonding interaction show an important role in stabilizing the reactant complex of tabun-inhibited enzyme with the charged oximes and thereby influencing the activation barrier. These calculated results suggest that the neutral oximes are kinetically more potent drug for the reactivation of tabun-inhibited AChE. Further, the calculated positive value of LogP indicates the hydrophilic nature of the neutral oximes suggesting they would be able to penetrate the blood-brain barrier. In particular, the weaker interaction of the neutral oximes inside the active-site gorge of AChE was augmented with a neutral peripheral site having aromatic hydrocarbon, connected through alkyl chains. The computational experiments performed to design an effective antidote for the reactivation of tabun-inhibited AChE yields oxime **2** as an effective system, which will attract the interest of experimentalists to examine its efficacy for the said purpose.

## Supporting Information

Figure S1 MP2/6-31+G*//M05-2X/6-31G* calculated energy profile diagram for the reactivation of tabun-inhibited ***m*** **AChE with 2-pyridinealdoxime in aqueous phase.**
(TIF)

Figure S2 M05-2X/6-31G* optimized geometries and selected bond distances (Å) for species involved in the reactivation process of tabun-conjugated serine (SUN) molecule with 2-pyridinealdoxime in aqueous phase. (red = oxygen; blue = nitrogen; white = hydrogen; yellow = phosphorus; gray = carbon).
(TIF)

Figure S3 **Stable conformation of tabun-inhibited AChE with Ortho-7 showing interaction with residues of peripheral anionic site and active site.** (Cyan = carbon, blue = nitrogen, white = hydrogen and red = oxygen) (Residues of peripheral anionic site are shown in tube format).
(TIF)

Figure S4 **Stable conformation of tabun-inhibited AChE with 2-PAM showing interaction with residues of peripheral anionic site and active site.** (Cyan = carbon, blue = nitrogen, white = hydrogen and red = oxygen) (Residues of peripheral anionic site are shown in tube format).
(TIF)

Figure S5 **Stable conformation of tabun-inhibited AChE with DZP showing interaction with residues of peripheral anionic site and active site.** (Cyan = carbon, blue = nitrogen, white = hydrogen and red = oxygen) (Residues of peripheral anionic site are shown in tube format).
(TIF)

Figure S6 **Stable conformation of tabun-inhibited AChE with 3-hydroxy-2-pyridinealdoxime showing interaction with residues of peripheral anionic site and active site.** (Cyan = carbon, blue = nitrogen, white = hydrogen and red = oxygen) (Residues of peripheral anionic site are shown in tube format).
(TIF)

File S1 M05-2X/6-31G* optimized Cartesian coordinates of all stationary points, including electronic energies and imaginary frequencies of transition states geometries are listed.
(DOC)

Table S1 **Binding energies (kcal/mol) calculated with MMFF force field of drug-tabun-conjugated-AChE complexes.**
(DOC)
