# Supplementary material for: In Silico Studies in Probing the Role of Kinetic and Structural Effects of Different Drugs for the Reactivation of Tabun-Inhibited AChE
Source: PLoS One. 2013 Dec 2;8(12):e79591. doi: 10.1371/journal.pone.0079591 (PMC3846473; doi:10.1371/journal.pone.0079591)
Supplement: File S1 — M05-2X/6-31G* optimized Cartesian coordinates of all stationary points, including electronic energies and imaginary frequencies of transition states geometries are listed. (DOC) [file pone.0079591.s007.doc]

**Supporting Information for**

**In Silico studies in Probing the role of kinetic and structural effects of different drugs for the reactivation of tabun-inhibited AChE**

Rabindranath Lo, Nellore Bhanu Chandar, Manoj K. Kesharwani, Aastha Jain, Bishwajit Ganguly*

**MP2/6-31+G*//M05-2X/6-31G* calculated Cartesian coordinates of all stationary points, including electronic energies and imaginary frequencies of transition states geometries. (All energies are given in Hartee).**

**2-PAM**

E= -454.82232

C -0.50262400 2.30935700 0.00012300

O 3.62077200 -0.22691100 -0.00010200

N 2.43006500 -0.61808300 0.00019100

C 1.49818600 0.34529200 -0.00011000

C 0.14252600 -0.05131800 -0.00003400

C -0.25905300 -1.41772300 0.00006400

C -2.57619600 -0.76198700 -0.00006500

C -2.17478800 0.53868400 -0.00005400

N -0.85592400 0.88592600 -0.00004500

C -1.57950300 -1.76460500 0.00001600

H 0.07903200 2.54127300 -0.89014000

H -1.41521700 2.89588300 0.00033500

H -2.87162500 1.36275300 -0.00002500

H -3.62876100 -0.99996300 -0.00011800

H -1.86265800 -2.80882000 0.00006400

H 0.52601800 -2.15714000 0.00017700

H 1.81748700 1.37927800 -0.00047700

H 0.07926300 2.54093100 0.89033600

**Tabun**

E= -1252.22947

N -0.01818000 4.18777300 -1.22194800

C -0.41469000 2.92313600 -0.64008500

C 0.76780500 1.96694700 -0.43593200

O 0.27238000 0.69833000 0.05901000

C -1.07848300 3.16073600 0.69950300

O -1.06650700 4.23477900 1.25496300

O 2.32503400 0.25609900 1.58901700

P 1.37608700 -0.35053300 0.62274100

N 0.35543500 -1.56638900 1.12392800

C -0.39739300 -1.26556500 2.35024800

C 0.90764300 -2.92770400 1.12074800

O 2.04759600 -0.92089000 -0.70739300

C 3.48378800 -1.16158500 -0.77389300

C 3.85381500 -1.25801900 -2.23523200

C -3.57671300 -2.54433900 -0.88783200

N -4.26008600 -1.35193100 -0.81259900

C -2.26320100 -2.24606600 -0.70252000

C -3.40552800 -0.35781200 -0.59180000

N -2.18975500 -0.88453600 -0.52259100

H -1.55359400 2.28534100 1.16681300

H 0.51525300 4.72040800 -0.54418100

H 0.57071600 4.03485100 -2.03221300

H -1.14118600 2.42612100 -1.29107200

H 1.47345300 2.38011600 0.28547800

H 1.27203500 1.79464600 -1.38730500

H -1.33063300 -0.36105600 -0.35476400

H -3.65710300 0.68255100 -0.48738000

H -1.38824100 -2.86903900 -0.68561600

H -5.25967000 -1.23733200 -0.90747800

H -4.07493900 -3.47974600 -1.06328200

H 3.62182700 -0.32407500 -2.74743800

H 4.92294600 -1.45231000 -2.32777900

H 3.30872500 -2.07075400 -2.71522900

H 3.69386900 -2.09037300 -0.24386500

H 3.99537400 -0.34163000 -0.27517600

H 0.22317500 -1.37493200 3.24409900

H -0.78693000 -0.24886200 2.30833100

H -1.24004000 -1.95469600 2.41354200

H 1.65212800 -3.07310700 1.90952900

H 0.08675700 -3.62495200 1.29071400

H 1.35258100 -3.14251700 0.15162700

**C1a**

E= -1707.06756

N -4.84774400 -1.17519600 3.07601300

C -3.76803800 -0.61501600 2.26956200

C -4.04767800 -0.90976000 0.80460300

O -2.97692100 -0.43316800 -0.03674100

C -3.60399500 0.86633100 2.54689300

O -3.58720800 1.73717100 1.70074000

O -1.58626100 -2.50108700 0.60908800

P -1.67825100 -1.35930800 -0.33778700

N -1.79612100 -1.61883200 -1.97495600

C -3.08782400 -2.09948300 -2.47537100

C -0.65586900 -2.30946100 -2.59237900

O -0.47993700 -0.30793700 -0.28690200

C 0.06876000 0.04936900 1.00960700

C 0.98712200 1.22977700 0.79477700

H -4.57540800 -1.24498600 4.04885800

H -2.83701700 -1.11698900 2.55045200

H -4.17836600 -1.98232000 0.68000300

H -4.94248800 -0.38994800 0.46593200

H -3.54758800 1.12925000 3.61529900

H -3.08151700 -2.00993000 -3.56171700

H -3.89293600 -1.48071700 -2.08460200

H -0.65836600 -2.08613900 -3.65979400

H -0.72714300 -3.39384800 -2.45847800

H 0.27716300 -1.96383500 -2.15207700

H -0.75457100 0.30931100 1.67856300

H 0.60489100 -0.81184200 1.40168100

H 1.43606600 1.52391900 1.74447000

H 0.43331300 2.07816900 0.39037100

H 1.78394100 0.95836200 0.10199800

C -1.52322200 3.15112600 -2.57970300

N -1.88644800 4.12074000 -1.67114700

C -1.90339500 1.95961600 -2.04423400

C -2.46694400 3.55144500 -0.61814800

N -2.48397000 2.24381000 -0.83104600

H -1.80245700 0.94833700 -2.40092000

H -2.85133000 4.05489900 0.25052000

H -2.87168900 1.56176200 -0.17665200

H -1.03545700 3.39040100 -3.50642100

H -5.67498400 -0.58998400 3.03288600

H -3.26835800 -3.14715100 -2.21345400

C 6.45250800 1.01215900 0.44942000

C 4.28866200 -0.14284200 1.66527900

C 4.52927400 -0.39683900 0.28636100

H 7.28450900 1.42928700 -0.09704800

H 3.42621500 -0.61601400 2.10754400

C 3.69085200 -1.22636600 -0.49637700

H 3.85361200 -1.41493800 -1.54951000

N 2.65008600 -1.80729800 0.11059800

O 1.93049100 -2.54046500 -0.61115900

H -1.73842500 5.11425400 -1.77720600

C 6.23678200 1.26883000 1.76950600

H 6.91264200 1.91377100 2.30959900

C 5.11741300 0.66818800 2.38793100

H 4.91535200 0.84973000 3.43501500

N 5.62477400 0.20422800 -0.27151500

C 5.92677100 -0.02366200 -1.68898400

H 6.08300200 -1.08664800 -1.86213800

H 5.10017100 0.33204700 -2.30110500

H 6.82855000 0.52265600 -1.94354400

**TS1a**

E= -1707.04899

1 Imaginary frequency = -97.19 cm^-1^

N -3.23252100 4.11368900 0.99811100

C -2.86838400 2.86054500 0.35197800

C -2.78209400 1.76171500 1.42162400

O -2.27672600 0.55496600 0.88093600

C -3.86025000 2.41433800 -0.69663900

O -3.58221900 1.73279700 -1.66232400

O -0.09107400 1.27414100 1.79560300

P -0.65378400 0.02124500 1.19662800

N -1.17679500 -1.35573300 2.00731600

C -1.93416400 -1.13318800 3.23529800

C -0.45853900 -2.62423100 2.01009700

O -0.43513900 -0.45003300 -0.33234200

C -0.28504900 0.56097900 -1.35261500

C 0.31200400 -0.11990600 -2.56417800

H -3.27764900 4.86242400 0.31647200

H -1.88313100 2.95326700 -0.10786300

H -2.13387000 2.13261900 2.21189300

H -3.77731300 1.56160600 1.83156000

H -4.90462900 2.72711300 -0.51456000

H -2.61948000 -1.97052300 3.38410700

H -2.52488500 -0.22286900 3.16184300

H -1.18871700 -3.41531700 2.20256800

H 0.31683700 -2.66881000 2.77895900

H 0.00109800 -2.79590200 1.04079900

H -1.26489300 0.98661300 -1.57222900

H 0.37064400 1.34720300 -0.97719200

H 0.43677000 0.59799300 -3.37610300

H -0.34380100 -0.92287800 -2.90543200

H 1.28469100 -0.54400900 -2.31148100

C -3.49159500 -3.26467000 -1.73085000

N -4.09089500 -2.38236000 -2.60192100

C -2.97008800 -2.51156300 -0.72525400

C -3.94451500 -1.13786500 -2.15054700

N -3.26850900 -1.20152900 -1.01281300

H -2.41700200 -2.77495200 0.15778800

H -4.30428200 -0.23798700 -2.61574600

H -2.98274700 -0.40357700 -0.42083100

H -3.48913600 -4.32518000 -1.90232300

H -4.15660100 4.04111300 1.41266900

H -1.27995700 -1.06309900 4.11120100

C 6.57911400 0.27322400 -0.80594000

C 3.95183500 0.98162300 -1.07504100

C 4.32452200 0.04435800 -0.09023100

H 7.59440100 -0.05265800 -0.64165800

H 2.90654900 1.23890900 -1.14486400

C 3.36932800 -0.56154900 0.79777600

H 3.65471500 -1.25661800 1.57666700

N 2.11205100 -0.24067400 0.63445400

O 1.29480900 -0.80197100 1.44653000

H -4.56994600 -2.62935200 -3.45558700

C 6.24416900 1.18436800 -1.76847100

H 7.01372200 1.60463600 -2.39748000

C 4.89295000 1.54316000 -1.90249000

H 4.59232700 2.26183400 -2.65264100

N 5.64283500 -0.28051900 0.00769100

C 6.08431800 -1.25774100 1.01682700

H 5.85525900 -0.88218200 2.01135000

H 5.58060700 -2.20598900 0.84648100

H 7.15543700 -1.39300300 0.91706000

**INa**

E= -1707.05060

N -3.45476300 4.07229200 -0.36786500

C -3.01077200 2.70109800 -0.58509200

C -2.81844000 2.01553600 0.77646300

O -2.18693300 0.76437800 0.62688200

C -3.99641300 1.87733600 -1.37789000

O -3.70743600 0.94783100 -2.10242800

O -0.07126600 1.93615600 1.18208400

P -0.50828500 0.50024600 1.15830400

N -0.95413300 -0.35241300 2.54753700

C -2.18938300 0.03335800 3.21528700

C -0.71450400 -1.79039600 2.62261400

O -0.33523700 -0.51102200 -0.11906000

C -0.28413100 0.08373100 -1.43566100

C 0.46208700 -0.87514400 -2.33832800

H -3.60156800 4.53911600 -1.25566500

H -2.04540900 2.69667300 -1.09316600

H -2.21521100 2.68741600 1.38403100

H -3.79041100 1.86866600 1.26212300

H -5.05577900 2.15870400 -1.22438700

H -2.14465300 -0.33549500 4.24221300

H -3.08073400 -0.37893700 2.72970500

H -1.56521800 -2.35901700 2.22600900

H -0.57647100 -2.07649400 3.66814500

H 0.18141900 -2.05297000 2.06868200

H -1.30171400 0.26460300 -1.78423400

H 0.23679400 1.03982100 -1.37013500

H 0.54013300 -0.45779200 -3.34353900

H -0.06069200 -1.83080400 -2.40163700

H 1.46536200 -1.04925900 -1.94747600

C -3.18001500 -3.69963300 -1.13228800

N -4.37614400 -3.07565600 -1.40413000

C -2.36943300 -2.75078800 -0.59046900

C -4.30235100 -1.79572000 -1.04609100

N -3.09313800 -1.58076800 -0.54895900

H -1.35291400 -2.79481500 -0.24630700

H -5.08050100 -1.06387200 -1.16006700

H -2.76069900 -0.67028100 -0.17045100

H -3.01496400 -4.73937500 -1.34631200

H -4.34975800 4.07785300 0.11201100

H -2.28046000 1.11799900 3.25464100

C 6.58591200 -0.11271200 -0.81251000

C 4.02896400 0.74836900 -1.25142200

C 4.34646400 0.07495900 -0.06144000

H 7.57172200 -0.47893200 -0.57227500

H 3.01009900 1.07946600 -1.38052000

C 3.35535600 -0.18382500 0.96482000

H 3.61171900 -0.61927200 1.92244300

N 2.13018000 0.13198500 0.69112100

O 1.28105900 -0.11065900 1.64719200

H -5.18883800 -3.50953000 -1.81737500

C 6.30654700 0.54073600 -1.98369900

H 7.09425100 0.70443500 -2.70299400

C 4.99471000 0.97798600 -2.20551900

H 4.73993300 1.49840400 -3.11826600

N 5.62737200 -0.33977200 0.11909100

C 6.00655500 -1.06295300 1.34739000

H 5.86029300 -0.41733300 2.20964900

H 5.40164000 -1.96152800 1.43703700

H 7.05285900 -1.33594100 1.27163500

**TS2a**

E= -1707.04848

1 Imaginary frequency = -638.70 cm^-1^

N 3.62589100 3.63824900 -1.62543700

C 3.01530900 2.64630700 -0.74479400

C 2.81104800 1.34795900 -1.52990000

O 2.13517200 0.39047500 -0.74618400

C 3.87413900 2.36356700 0.46776200

O 3.47678200 2.30167700 1.61008300

O 0.01445800 0.95395900 -1.94765400

P 0.31643100 -0.24340200 -1.10377100

N 0.92278800 -1.67180700 -1.73546800

C 2.11691800 -1.63653900 -2.56482900

C 0.78244200 -2.90879800 -0.97130800

O 0.21022000 -0.29486500 0.51796600

C 0.21042800 0.94433900 1.26470100

C -0.58731900 0.70892200 2.52942900

H 3.74906900 4.51653400 -1.13448400

H 2.03963300 2.99866200 -0.40724100

H 2.21682100 1.58387200 -2.41035700

H 3.78044500 0.94700800 -1.85361700

H 4.94776000 2.21506700 0.23687400

H 2.08665100 -2.49621500 -3.23746500

H 3.04129000 -1.68293000 -1.97895000

H 1.63075200 -3.06328300 -0.29502900

H 0.73739300 -3.74924100 -1.66727200

H -0.13589700 -2.88841300 -0.39128900

H 1.24095200 1.22528800 1.47641300

H -0.24633000 1.72479800 0.65443000

H -0.61616000 1.62195900 3.12620000

H -0.13274900 -0.08055200 3.13027700

H -1.60785000 0.41472300 2.28150900

C 3.82827600 -2.31216000 2.58820600

N 4.98909700 -2.07898300 1.89114000

C 2.84779700 -1.64839500 1.91215100

C 4.71054000 -1.30435400 0.83495600

N 3.41790600 -1.02965100 0.82401300

H 1.79283900 -1.57424200 2.10829800

H 5.43805700 -0.96545200 0.11761600

H 2.80946300 -0.31806700 0.02159000

H 3.80585900 -2.91381200 3.47855700

H 4.55192100 3.33174500 -1.90857300

H 2.12723500 -0.73271100 -3.17154300

C -6.76280800 0.17370600 0.63204800

C -4.25405800 1.25662800 0.59319800

C -4.49111500 0.01241500 0.00233000

H -7.72646900 -0.31045800 0.60935100

H -3.25135200 1.65292200 0.54742600

C -3.42760400 -0.74708600 -0.64987300

H -3.62341500 -1.66730200 -1.18553700

N -2.23667300 -0.27592000 -0.56574000

O -1.33133300 -1.01708400 -1.19219400

H 5.90406000 -2.43066600 2.12917900

C -6.56454600 1.39630600 1.22481800

H -7.39767500 1.90210600 1.68831600

C -5.28157800 1.94707100 1.20470100

H -5.09181800 2.90902400 1.65989600

N -5.74835100 -0.49670500 0.03943400

C -6.04263300 -1.80986600 -0.57031400

H -5.85111200 -1.76296600 -1.63909300

H -5.42542400 -2.57121800 -0.10123500

H -7.08882000 -2.03484300 -0.39910200

**C2a**

E= -1707.07354

N -3.75961000 3.62353900 -2.15827100

C -3.42071500 3.10561200 -0.82998300

C -3.34018200 1.57970900 -0.77595200

O -3.02474100 1.17620300 0.53841300

C -2.08238300 3.70114600 -0.45601900

O -1.85007900 4.25723800 0.59434600

O -0.02483700 1.38651400 -0.76866200

P -0.07715100 0.14248300 0.02978600

N -0.08605700 0.30187700 1.65613000

C -0.33947400 -0.87869500 2.47878400

C -0.45321500 1.57651800 2.27127300

O -1.17379300 -0.95047300 -0.32968700

C -1.40420700 -1.36743000 -1.70512100

C -0.84395900 -2.75554400 -1.92624900

C -4.58759300 -3.61593700 0.65369500

N -5.74685600 -2.92128300 0.41676900

C -3.61005700 -2.66420100 0.75291200

C -5.44126100 -1.60571600 0.38266700

N -4.15426700 -1.41189300 0.58235600

C 5.71306800 -1.97800300 -1.55878800

O 1.25114300 -0.84072900 -0.27489300

N 2.42292000 -0.17771400 -0.00363000

C 3.43403800 -0.89506700 -0.28964700

C 4.75909200 -0.32013200 -0.00519300

C 4.91340500 0.74586100 0.87187300

C 7.27943800 0.69792600 0.49104400

C 7.08286100 -0.36095500 -0.37014400

N 5.85117500 -0.85087200 -0.60959300

C 6.17572500 1.26411500 1.11989000

H 5.02031600 -1.69848000 -2.34741900

H 6.68793400 -2.17848300 -1.98743000

H 7.89644500 -0.84038100 -0.89129500

H 8.28176900 1.06193600 0.65769400

H 6.29638700 2.09410400 1.80150900

H 4.03239400 1.14497500 1.35052700

H 3.32083100 -1.88885600 -0.70632900

H -1.30522800 3.60488800 -1.23129700

H -3.17486300 3.16845700 -2.85369900

H -4.71354400 3.36134600 -2.38220300

H -4.15457400 3.45585500 -0.10334400

H -2.57637400 1.24902100 -1.49147300

H -4.30709900 1.17501800 -1.09900200

H -3.28679300 0.22927400 0.61294800

H -6.17947300 -0.83940000 0.21239400

H -2.55682600 -2.79648900 0.93384400

H -6.66408100 -3.31829600 0.28943600

H -4.56637400 -4.68864700 0.72792800

H -1.30678000 -3.46335900 -1.23813500

H -1.05793200 -3.07489200 -2.94760300

H 0.23477900 -2.76368900 -1.77385100

H -0.95762500 -0.63534800 -2.37856200

H -2.48503900 -1.34603600 -1.82659700

H -1.41220200 -1.02652100 2.63610200

H 0.07653700 -1.76552200 2.00311100

H 0.14784900 -0.74246100 3.44461600

H -1.51420900 1.59528000 2.52405900

H 0.14799000 1.70950300 3.17237400

H -0.25046800 2.38721100 1.57757200

H 5.36335000 -2.85812200 -1.02537400

**Ortho-7**

E= -1104.32126

C -3.03515400 -0.64579800 0.47469200

C -1.11014200 -1.92854000 1.45101400

O -4.02485000 3.99312400 -0.40347900

N -4.74206900 2.97475300 -0.54478700

C -4.17099200 1.80810600 -0.21298600

C -4.94375500 0.63248100 -0.35939700

C -6.27983800 0.66312900 -0.84801400

C -6.43248500 -1.72726600 -0.65240400

C -5.15332200 -1.73854800 -0.18213900

N -4.42579300 -0.59662900 -0.03353000

C -2.52518000 -2.02417300 0.86737900

C -7.00747300 -0.48368700 -0.99177600

C -0.05644100 -1.41713000 0.46716800

C 1.35857700 -1.55096100 1.02783400

C 2.41516800 -1.01423900 0.06577800

C 3.81395300 -1.20696200 0.63842000

N 4.83693700 -0.58573800 -0.22491400

C 5.41409600 -1.39464500 -1.15877700

C 6.36014700 -0.95012000 -2.03211400

C 6.74432500 0.40503000 -1.95115600

C 6.15690800 1.21873200 -1.02396000

C 5.15787100 0.74287700 -0.12649500

C 4.60481700 1.67955600 0.78357000

N 3.59017300 1.45720300 1.62502000

O 3.25684000 2.44136100 2.33327800

H -2.40513300 -0.21845100 -0.30539500

H -2.99168600 0.01418200 1.34279700

H -3.18226900 -2.46653300 1.61932300

H -2.50676000 -2.68788500 -0.00108200

H -1.12299300 -1.29012500 2.33990000

H -0.82126600 -2.92780800 1.78740600

H -0.23753900 -0.36619600 0.22230200

H -0.13120200 -1.97907800 -0.47047900

H 1.43015000 -1.00466400 1.97410600

H 1.56194400 -2.60378400 1.25097800

H 2.24668200 0.05018800 -0.10886800

H 2.34616900 -1.52817200 -0.89800300

H 3.88950900 -0.75293100 1.62111000

H 4.06154400 -2.26486800 0.71116600

H 5.06670500 -2.41715400 -1.15185700

H 6.79180600 -1.62896200 -2.75138500

H 7.49594100 0.80032400 -2.62092100

H 6.43094600 2.26141500 -0.95052400

H 5.03008100 2.67892600 0.76035700

H -4.66031800 -2.65477000 0.09215100

H -6.97245700 -2.65596400 -0.75434900

H -8.02118000 -0.43701400 -1.36660900

H -6.68449900 1.63036800 -1.09941700

H -3.15132200 1.81890800 0.14655500

**C1b**

E= -2356.56595

N 7.72477300 -2.70769100 -2.15896500

C 6.64636600 -2.06999800 -1.42195300

C 5.53565800 -3.10881600 -1.21707800

O 4.42829600 -2.58008400 -0.47664400

C 7.05431900 -1.51595000 -0.07530300

O 6.46938800 -0.61285900 0.48696200

O 3.66246100 -1.45652900 -2.65524500

P 3.36335200 -1.58789500 -1.20675400

N 1.88585100 -2.19232900 -0.78869000

C 1.72539800 -3.62952600 -1.01284700

C 1.10614900 -1.66258700 0.32651900

O 3.49095700 -0.23336400 -0.35394600

C 3.92624700 0.98463400 -1.03986600

C 3.87665200 2.11008900 -0.03396900

H 8.41981900 -2.02267100 -2.43056100

H 6.23277300 -1.25452500 -2.01830600

H 5.21431300 -3.45192100 -2.19855100

H 5.90404800 -3.95582600 -0.63901400

H 7.92597300 -2.00027800 0.39773100

H 0.66810600 -3.84436100 -1.16925300

H 2.08963100 -4.21472300 -0.16309400

H 1.46628000 -2.03363700 1.29235100

H 0.07311200 -1.98785700 0.19659400

H 1.12737500 -0.57741400 0.29752400

H 4.93888800 0.82130400 -1.40428600

H 3.24401700 1.15695100 -1.86567700

H 4.13839900 3.04549700 -0.53115700

H 4.58571000 1.94517600 0.77860700

H 2.87013300 2.20840600 0.37376300

C 3.50748000 -0.06571000 4.32221200

N 4.84627000 -0.35886800 4.44917300

C 3.23378800 -0.09763200 2.99046900

C 5.37812200 -0.56237400 3.24698700

N 4.41078200 -0.41158700 2.35300300

H 2.32182600 0.07832300 2.45037500

H 6.40425200 -0.79649400 3.03024400

H 4.53278300 -0.49943600 1.34317700

H 2.88230200 0.13770700 5.17196400

H 8.19672300 -3.39495000 -1.58015200

H 2.26760900 -3.93186200 -1.90928400

C -0.07712300 2.62467600 -0.88683700

H -0.89755800 2.00451700 -0.54890800

N 0.92643400 2.05146700 -1.55524600

O 0.83748600 0.80878200 -1.72576400

H 5.35737200 -0.41144200 5.31890000

C -2.17684000 3.96249600 0.50881900

C -4.45799600 3.01858600 -0.07843600

C -0.01051100 4.02532800 -0.67784200

C 1.07593100 4.80941500 -1.15681800

C 0.06501200 6.80607100 -0.26847400

C -0.96224800 6.03845200 0.18984700

N -0.99684200 4.68848300 0.00113600

C -3.21048000 3.74046000 -0.59329700

C 1.11490400 6.16104000 -0.95957800

C -4.18503200 1.60667300 0.44324400

C -5.47513500 0.85041400 0.75968300

C -5.19235000 -0.52125800 1.36858300

C -6.45013300 -1.31088300 1.72076700

N -7.20021100 -1.71233500 0.51265900

C -8.25012300 -0.92058000 0.14847300

C -8.99883100 -1.15693300 -0.96499300

C -8.65888200 -2.26977800 -1.76248900

C -7.61021600 -3.06529200 -1.39696900

C -6.83691700 -2.80754000 -0.22845100

C -5.78545300 -3.71458600 0.05934400

N -4.92222400 -3.63305100 1.07670800

O -4.07856700 -4.56364500 1.13769800

H -1.82980300 3.03283200 0.95222300

H -2.60186300 4.56529500 1.30976200

H -3.48843300 4.71636200 -0.99937700

H -2.76115300 3.16786700 -1.40868800

H -4.92716900 3.61378200 0.71179800

H -5.18029300 2.96088500 -0.89737300

H -3.57264300 1.64792900 1.34942600

H -3.61092000 1.04463200 -0.30230700

H -6.08201800 1.44335100 1.45318900

H -6.05767600 0.74353500 -0.16141900

H -4.62056200 -0.40070100 2.29317100

H -4.57568400 -1.11747800 0.69305400

H -6.19437600 -2.21532800 2.26126100

H -7.12794300 -0.70908600 2.32427700

H -8.44636400 -0.09062800 0.81059600

H -9.82165800 -0.50317200 -1.20991200

H -9.22426000 -2.49467700 -2.65660500

H -7.33550900 -3.92448600 -1.99186100

H -5.67351200 -4.54833400 -0.62797000

H -1.80278400 6.44951000 0.72891600

H 0.06089000 7.87124000 -0.09589400

H 1.95037300 6.73663500 -1.33508400

H 1.85865800 4.28761600 -1.68427600

**TS1b**

E= -2356.55387

1 Imaginary frequency = -128.73 cm^-1^

N -6.10894900 -1.27706800 4.12069000

C -5.82141400 -1.02066000 2.71651600

C -4.71242000 -1.97619900 2.25393800

O -4.24870800 -1.62432400 0.96133100

C -7.01279200 -1.22373900 1.81001800

O -7.16782100 -0.67351700 0.73942700

O -2.53675000 -0.22568300 2.11817400

P -2.74327200 -0.80911500 0.75893000

N -1.85967700 -2.11379200 0.18837700

C -2.20174100 -3.42423500 0.73166700

C -1.45637900 -2.17240000 -1.21313400

O -3.32118300 0.02826500 -0.50246000

C -4.21200500 1.13503500 -0.20545600

C -4.19131800 2.06636300 -1.39677300

H -6.82538500 -0.64220700 4.45400700

H -5.44604800 -0.00372900 2.59227600

H -3.91353100 -1.91331900 2.98997100

H -5.08432400 -3.00430900 2.21313300

H -7.75077400 -1.96321300 2.17151200

H -1.35518800 -4.09090800 0.55866900

H -3.09440900 -3.85359400 0.26462400

H -2.23290500 -2.63692600 -1.83316400

H -0.55043200 -2.77678600 -1.29517900

H -1.24494600 -1.17374500 -1.58102200

H -5.20905600 0.73532000 -0.01379900

H -3.85340500 1.63824300 0.69173200

H -4.84088100 2.92286500 -1.20902300

H -4.54369400 1.55522300 -2.29385800

H -3.17462300 2.42175200 -1.56802000

C -5.73500500 -1.67799300 -3.76484300

N -6.92014200 -2.05964000 -3.17843000

C -4.87142600 -1.41837700 -2.74657100

C -6.79299900 -2.03408700 -1.85430500

N -5.55563500 -1.64849100 -1.57458500

H -3.85238300 -1.07879400 -2.74802000

H -7.56130800 -2.26131400 -1.13848100

H -5.16669900 -1.56227700 -0.62356500

H -5.61457500 -1.62214200 -4.83097500

H -6.47626500 -2.21633000 4.23915900

H -2.36195600 -3.36275000 1.80766000

C -0.00555000 2.21037000 -0.04846000

H 0.94253900 1.71857000 -0.22473500

N -1.07614700 1.48869800 0.17815300

O -0.89686000 0.22330800 0.16929700

H -7.76655000 -2.31360300 -3.66717100

C 2.21286700 3.89011000 -0.69418800

C 4.38217400 2.96848600 0.24044700

C -0.15802600 3.63935900 -0.03548000

C -1.38166100 4.25577300 0.30635000

C -0.39723700 6.42744100 -0.00029300

C 0.77496400 5.80767700 -0.32824800

N 0.88707500 4.45281500 -0.35503600

C 3.00565700 3.55284500 0.56683300

C -1.50380000 5.62236900 0.32154400

C 4.32398300 1.60195600 -0.44364300

C 5.71163400 0.98362800 -0.61654700

C 5.64596400 -0.36788500 -1.32480900

C 7.01295800 -0.99445100 -1.58428900

N 7.70030800 -1.35258100 -0.32650400

C 8.60626100 -0.45280700 0.15504500

C 9.28268800 -0.64191300 1.32249700

C 9.02140300 -1.82175700 2.05006500

C 8.11605500 -2.72394200 1.56830700

C 7.41649400 -2.51392700 0.34498300

C 6.51495000 -3.52984700 -0.06241800

N 5.73354100 -3.50920500 -1.14679600

O 5.02208300 -4.53273200 -1.31264500

H 2.05430900 3.03037600 -1.33907800

H 2.73467700 4.64374900 -1.28068200

H 3.11894200 4.46947100 1.15086600

H 2.44082700 2.84672100 1.18154300

H 4.94045800 3.67032600 -0.38772400

H 4.94057400 2.87277500 1.17545600

H 3.85505900 1.68614600 -1.42899400

H 3.69840400 0.92476400 0.14884600

H 6.34462000 1.67115100 -1.18890700

H 6.17364700 0.87183900 0.36982100

H 5.16016100 -0.24922800 -2.29759700

H 5.03599200 -1.06816700 -0.75091400

H 6.91212300 -1.90069200 -2.17119500

H 7.66386200 -0.29561300 -2.10748500

H 8.74932400 0.42331100 -0.45987800

H 9.99176700 0.09797200 1.66053800

H 9.53424300 -2.01356800 2.98271500

H 7.90352600 -3.63507000 2.10882400

H 6.45316500 -4.40065300 0.58400600

H 1.67023900 6.35280900 -0.58647800

H -0.45068300 7.50528000 0.00611700

H -2.44805500 6.07706400 0.58830400

H -2.20567500 3.60908000 0.56361400

**INb**

E= -2356.55561

N -6.11507800 -1.21783800 4.06700900

C -5.81919400 -0.98786900 2.65843000

C -4.60758000 -1.84284900 2.25486300

O -4.14565600 -1.49146500 0.97030400

C -6.96329100 -1.35175900 1.74253500

O -7.19586300 -0.82670000 0.67379000

O -2.46612400 -0.07826600 2.12439800

P -2.60537200 -0.62817000 0.73446800

N -1.78469100 -2.00038500 0.19021600

C -2.15784700 -3.29001900 0.75381200

C -1.39624500 -2.10355400 -1.21311200

O -3.31198700 0.16013000 -0.51523100

C -4.32250100 1.14246800 -0.19549200

C -4.38978900 2.11910900 -1.35035500

H -6.92129000 -0.67268400 4.35098400

H -5.55036200 0.05754100 2.50016600

H -3.83881400 -1.67288300 3.00594500

H -4.88139600 -2.90477400 2.25664500

H -7.58770100 -2.19239100 2.10093700

H -1.32648100 -3.97966900 0.59248600

H -3.05920000 -3.70987200 0.29370200

H -2.18316300 -2.58026000 -1.81126100

H -0.49478000 -2.71602500 -1.29221800

H -1.18599600 -1.11916200 -1.61945300

H -5.27442800 0.63482300 -0.03639300

H -4.03905000 1.65042100 0.72740100

H -5.12674000 2.89592700 -1.13996400

H -4.68009800 1.60974800 -2.27059800

H -3.41574900 2.58581100 -1.50138500

C -5.59122500 -1.84521300 -3.68660300

N -6.63104800 -2.50771500 -3.07538200

C -4.76867200 -1.42376900 -2.68831400

C -6.45517300 -2.49183500 -1.75582100

N -5.32936300 -1.84057300 -1.50249900

H -3.85405200 -0.86109600 -2.70844700

H -7.12004200 -2.91859900 -1.02765000

H -4.93585200 -1.68106600 -0.55095100

H -5.53151400 -1.73238600 -4.75338200

H -6.35722400 -2.19201300 4.22057400

H -2.31798800 -3.20687000 1.82794000

C -0.07277700 2.25561200 -0.08852000

H 0.86236100 1.74726300 -0.28688200

N -1.14261400 1.57465200 0.16892700

O -0.97902400 0.28290000 0.15773500

H -7.41530000 -2.93743300 -3.54425700

C 2.17983400 3.89082800 -0.75937100

C 4.33587500 2.95925600 0.19431900

C -0.19639500 3.70263300 -0.08624900

C -1.40034400 4.33680800 0.26131200

C -0.38223100 6.48444700 -0.08236800

C 0.77782200 5.83777800 -0.41603800

N 0.86244600 4.48400300 -0.42502800

C 2.96204300 3.55628000 0.50868400

C -1.49759600 5.71003000 0.26201000

C 4.27274900 1.58932800 -0.48233500

C 5.65733600 0.95828600 -0.63255300

C 5.59067500 -0.39897900 -1.32942400

C 6.95618200 -1.04143700 -1.55689400

N 7.61564300 -1.39263500 -0.28234800

C 8.52209200 -0.49720100 0.20637500

C 9.17240700 -0.67897400 1.38962700

C 8.88274600 -1.84636000 2.12663200

C 7.97701400 -2.74431100 1.63783300

C 7.30501800 -2.54218900 0.39777600

C 6.40057400 -3.55314400 -0.01542900

N 5.64581700 -3.53965500 -1.11868600

O 4.92725300 -4.55767700 -1.28733900

H 2.00662300 3.02853800 -1.39629300

H 2.71336000 4.62984500 -1.35329700

H 3.07935900 4.47589300 1.08698100

H 2.38883000 2.85867200 1.12553600

H 4.90424400 3.65372900 -0.43274000

H 4.88543800 2.86472000 1.13456300

H 3.81702500 1.67153600 -1.47405800

H 3.63428900 0.92128100 0.10675200

H 6.30339800 1.63581700 -1.20216400

H 6.10496600 0.85166100 0.36095400

H 5.12392400 -0.28506200 -2.31200300

H 4.96349300 -1.08767900 -0.76002100

H 6.85730700 -1.95270200 -2.13617000

H 7.62381800 -0.35456700 -2.07486000

H 8.68793600 0.36930700 -0.41634700

H 9.88313700 0.05701200 1.73267100

H 9.37450700 -2.03188200 3.07183500

H 7.74320500 -3.64611000 2.18516900

H 6.31338700 -4.41382500 0.64160300

H 1.67932700 6.36418400 -0.69030500

H -0.41355100 7.56319600 -0.09080300

H -2.42993700 6.18574900 0.53292800

H -2.23425600 3.70811800 0.53240200

**TS2b**

E= -2356.55367

1 Imaginary frequency = -702.97 cm^-1^

N -6.80973600 -0.99801000 3.57788200

C -6.24507500 -0.70884100 2.26279000

C -4.97540100 -1.54472000 2.07900400

O -4.33890100 -1.23210500 0.85998800

C -7.21734400 -1.02762800 1.14884900

O -7.46617100 -0.30734100 0.20757100

O -2.71100300 0.18339900 2.12435100

P -2.59715200 -0.34162300 0.72848500

N -1.86133800 -1.80264100 0.36115400

C -2.28742200 -3.00929300 1.05271500

C -1.41724300 -2.04865200 -1.00868100

O -3.25506400 0.35059100 -0.58684700

C -4.33997000 1.29366800 -0.42229400

C -4.22493400 2.31466900 -1.53395100

H -7.63948200 -0.43767500 3.73624600

H -5.97521500 0.34605800 2.19757200

H -4.30632500 -1.31596400 2.90641000

H -5.22306500 -2.61396200 2.10650300

H -7.72015400 -2.00930900 1.25489200

H -1.45044400 -3.71054900 1.05474300

H -3.14672200 -3.49064600 0.57333700

H -2.22005500 -2.47007700 -1.62432500

H -0.58956800 -2.76066700 -0.98547200

H -1.06820700 -1.12502500 -1.46195200

H -5.28302300 0.75033800 -0.45904700

H -4.25170700 1.76913300 0.55537500

H -5.02955200 3.04708300 -1.45069400

H -4.29860600 1.83164500 -2.50973600

H -3.26634400 2.83074300 -1.47140300

C -5.49450900 -2.57572300 -3.40403100

N -6.05783500 -3.60577900 -2.69040000

C -4.90578100 -1.76174100 -2.48191900

C -5.81213200 -3.41399700 -1.38807900

N -5.11418200 -2.30236800 -1.23425200

H -4.34737100 -0.85167600 -2.61149600

H -6.14156300 -4.07101100 -0.60177800

H -4.74016600 -1.79608600 -0.17281300

H -5.56133400 -2.51713300 -4.47517300

H -7.10141000 -1.96971300 3.62705300

H -2.54081300 -2.78042400 2.08647500

C -0.03401500 2.37930500 0.02534400

H 0.88859000 1.82310900 -0.08583200

N -1.14164100 1.77022500 0.24442300

O -1.01322700 0.45020500 0.30776200

H -6.57120600 -4.38381100 -3.07616900

C 2.25051800 3.84542100 -0.88694800

C 4.43260300 2.88143000 -0.03375300

C -0.08339300 3.83941700 -0.05001000

C -1.22588400 4.55033800 0.33014500

C -0.12097300 6.61385700 -0.19911200

C 0.98331400 5.88434700 -0.56324900

N 0.99302400 4.53303200 -0.49797200

C 3.12225400 3.57915100 0.33830400

C -1.25114400 5.92877100 0.25279800

C 4.25019500 1.44194000 -0.51665900

C 5.58945600 0.74557300 -0.75903500

C 5.40512000 -0.69847500 -1.22084600

C 6.71429800 -1.41328200 -1.54301800

N 7.56350000 -1.57119600 -0.34422200

C 8.53927300 -0.63541000 -0.15790700

C 9.36522300 -0.63808500 0.92514300

C 9.18911200 -1.65735800 1.88441500

C 8.21450400 -2.59605300 1.69832100

C 7.35806600 -2.58205400 0.55940300

C 6.39668500 -3.62020500 0.46539400

N 5.48001500 -3.76778900 -0.49642300

O 4.74261000 -4.77959600 -0.38050200

H 1.98385000 2.94452900 -1.43117500

H 2.76140000 4.50567900 -1.58411700

H 3.33358600 4.53785000 0.81760800

H 2.57328700 2.97211600 1.06389600

H 4.95542900 3.46497500 -0.79837200

H 5.07486100 2.87775800 0.85054600

H 3.66917900 1.41589700 -1.44399700

H 3.68098300 0.87864100 0.23122500

H 6.15687200 1.30626500 -1.51028700

H 6.17292700 0.77369500 0.16710300

H 4.79891100 -0.71933300 -2.13105100

H 4.86236700 -1.27111400 -0.46636700

H 6.52069700 -2.40350700 -1.94013700

H 7.29711600 -0.84279100 -2.26456600

H 8.61040200 0.11018900 -0.93569100

H 10.12417900 0.12232000 1.02620600

H 9.82155400 -1.69794700 2.76083700

H 8.06477600 -3.38703200 2.41904700

H 6.41029400 -4.35762300 1.26311800

H 1.89189300 6.34327800 -0.92254500

H -0.09351500 7.69054700 -0.26919000

H -2.13814400 6.47107200 0.54901100

H -2.07290900 3.98675900 0.69018300

**C2b**

E= -2356.57820

N 4.47875800 -2.84342200 3.39856800

C 4.81677700 -2.12084800 2.17005000

C 3.89390200 -2.46860000 0.99041700

O 4.17817800 -1.72663000 -0.16960300

C 4.73499000 -0.64460400 2.47632700

O 5.55751300 0.17373000 2.12187500

O 2.31718100 0.40200200 1.16243100

P 1.91783100 0.29739600 -0.25896800

N 2.95617700 0.89065600 -1.36557600

C 2.65525700 0.76806700 -2.78806500

C 4.35626600 1.14464600 -1.04214200

O 1.38335600 -1.10248400 -0.79704200

C 0.20912300 -1.71722300 -0.19175700

C 0.21408000 -3.17868300 -0.57394500

C 9.02551300 -1.99969200 -1.54440100

N 8.92471400 -1.06247100 -0.54659200

C 7.77308300 -2.53503200 -1.66437900

C 7.64876400 -1.05921600 -0.10483500

N 6.92164600 -1.94261400 -0.76074800

C -2.80337500 4.22158700 0.86625600

C -4.76700300 2.80022200 0.17258900

O 0.48886000 1.12867300 -0.55453200

N 0.60940800 2.47298600 -0.29374400

C -0.53142400 3.03485000 -0.33004800

C -0.57454500 4.49782500 -0.16211400

C 0.47710700 5.30414700 -0.57420300

C -0.76036500 7.23972300 0.11778400

C -1.77881100 6.40054600 0.52016400

N -1.67608400 5.06495600 0.38947800

C -3.77670000 3.88570900 -0.26319600

C 0.39144700 6.68203200 -0.42694200

C -4.12564400 1.41822700 0.31500300

C -5.12931600 0.34883000 0.74490600

C -4.45256200 -1.00515700 0.95215800

C -5.39135500 -2.08766400 1.47571900

N -6.41571400 -2.45628500 0.47716000

C -7.62801300 -1.84081200 0.58639300

C -8.64963700 -2.06786400 -0.28647800

C -8.42546300 -2.97938800 -1.33841100

C -7.21189100 -3.59889900 -1.44620100

C -6.15322600 -3.35076700 -0.52738500

C -4.94392700 -4.06737700 -0.72856900

N -3.81657000 -3.93088900 -0.02812800

O -2.87647300 -4.69369000 -0.37774300

H -2.36806300 3.34020300 1.32896100

H -3.30317900 4.78370200 1.65104600

H -4.30688400 4.79755400 -0.54367400

H -3.23268400 3.55202600 -1.15116000

H -5.23483100 3.08925200 1.11921800

H -5.56731800 2.74652300 -0.56909100

H -3.31766900 1.44136400 1.05386900

H -3.67457100 1.12637900 -0.64020500

H -5.61274600 0.66390700 1.67631500

H -5.91555400 0.27064700 -0.01294800

H -3.64695300 -0.90167600 1.68488700

H -3.99208700 -1.34986100 0.02390500

H -4.83811300 -2.98693700 1.72203300

H -5.92631000 -1.74063700 2.35852400

H -7.72038100 -1.16048300 1.41974500

H -9.59210100 -1.55860600 -0.15702500

H -9.20759100 -3.18972200 -2.05517300

H -7.02026200 -4.30293600 -2.24315900

H -4.94485100 -4.78350200 -1.54545900

H -2.69302700 6.76536600 0.96368100

H -0.87609600 8.30604000 0.23917500

H 1.20911100 7.31278900 -0.74571000

H 1.34152400 4.83287700 -1.01656000

H -1.43156700 2.45946500 -0.51704000

H 3.86442600 -0.34146300 3.07671100

H 3.47366500 -2.80449300 3.54366100

H 4.70476800 -3.82467900 3.27855800

H 5.84987900 -2.34365900 1.89650800

H 2.86346300 -2.24233100 1.27264300

H 3.96924200 -3.55094700 0.81962700

H 5.11842200 -1.88351600 -0.41938600

H 7.28664100 -0.42086900 0.68627100

H 7.43765000 -3.30376900 -2.34051200

H 9.67050200 -0.48028900 -0.20078000

H 9.94931600 -2.19260600 -2.06002300

H 1.08038800 -3.68222000 -0.14305700

H -0.70024500 -3.66054600 -0.22344300

H 0.25745700 -3.27616300 -1.65976200

H -0.67237700 -1.20289800 -0.57296200

H 0.26191900 -1.58231700 0.88940300

H 3.13172100 -0.11966600 -3.21293000

H 1.57988400 0.70322800 -2.94355500

H 3.02753900 1.65385300 -3.30480300

H 4.99707100 0.38383300 -1.49269300

H 4.63425000 2.12787700 -1.42782600

H 4.49557800 1.11822300 0.03451700

**DZP**

E= -438.35023

C 0.51704000 2.40120200 -0.00019100

O -3.68898400 -0.22163600 0.00017000

N -2.46215200 -0.62788300 -0.00008400

C -1.53062800 0.29198400 0.00006500

C -0.11779100 -0.06245300 0.00005400

C 0.29602900 -1.40907600 -0.00020700

C 2.62177000 -0.77154400 0.00017900

C 2.22805100 0.56410500 0.00016000

C 1.63827500 -1.76013900 -0.00009000

H -0.07659100 2.66693400 0.87849400

H 1.41478100 3.02025900 -0.00116200

H 2.98205400 1.34454700 0.00017000

H 3.67249700 -1.03348700 0.00033500

H 1.91804100 -2.80747900 -0.00021600

H -0.46975800 -2.17335400 -0.00052900

H -1.83413200 1.33754400 0.00016400

H -0.07798000 2.66618800 -0.87816100

C 0.88359200 0.93710600 0.00005200

**C1c**

E= -1690.58827

N -5.87197900 -2.75878200 -0.34557300

C -4.73130800 -1.93444100 -0.73517700

C -4.16777300 -1.12273300 0.43173300

O -3.08797600 -0.27651600 -0.01654600

C -3.67959600 -2.86449100 -1.30688100

O -3.09583700 -2.65649100 -2.34570500

O -1.42185700 -2.16792200 0.49406500

P -1.54592300 -0.69542500 0.32584500

N -0.77642600 0.15502900 -0.85488600

C 0.64513400 0.47822800 -0.69339500

C -1.19922600 0.04953300 -2.25077700

O -1.17621000 0.15062700 1.62893100

C -0.95845100 -0.51112400 2.91013800

C -0.33358200 0.51057000 3.83046200

C -1.66013100 4.41100600 -0.04364000

N -2.72616200 4.65976300 -0.87875900

C -1.71674100 3.08932500 0.27015000

C -3.41470200 3.53897600 -1.07288300

N -2.81560500 2.57809000 -0.38257400

C 5.16504800 2.10121000 -0.22288600

O 1.83405100 -0.87199800 1.90863200

N 2.91169500 -1.17545800 1.24621500

C 3.64382000 -0.18134700 0.82704900

C 4.87136800 -0.40944700 0.06880100

C 5.34358100 -1.71191300 -0.17680400

C 7.24421200 -0.86543500 -1.39424200

C 6.78810800 0.42872800 -1.15847600

C 6.50834600 -1.93990300 -0.89623500

H 5.12386400 2.35452400 0.83969200

H 5.84930900 2.79747200 -0.70869200

H 7.35024600 1.27322000 -1.54310600

H 8.15546300 -1.03082100 -1.95523000

H 6.84342300 -2.95632600 -1.06679100

H 4.76953600 -2.54194100 0.21312000

H 3.32593000 0.83516800 1.05409900

H -3.49453400 -3.77840800 -0.71862900

H -5.65767200 -3.27452300 0.50238200

H -6.66735400 -2.16743000 -0.13298400

H -5.02956500 -1.25289900 -1.53262800

H -3.81867700 -1.78738100 1.22291800

H -4.94048300 -0.46618200 0.82739500

H -3.10985800 1.60029100 -0.34593700

H -4.29547000 3.43362500 -1.68051300

H -1.09131100 2.47030800 0.88902700

H -2.95981400 5.55314100 -1.28817100

H -0.97043700 5.18127100 0.24799300

H -0.96600300 1.39532700 3.91802300

H -0.20393600 0.07630700 4.82331000

H 0.64319400 0.78509600 3.43555800

H -0.28596300 -1.34645300 2.74659100

H -1.92464400 -0.85699000 3.27873500

H 0.81015800 1.50947500 -1.01633900

H 0.95347300 0.34876800 0.34428500

H 1.25643900 -0.18464200 -1.31235000

H -1.04567700 1.01788900 -2.73172900

H -0.61117500 -0.70394900 -2.78275400

H -2.24987200 -0.21921300 -2.31650600

H 4.16428000 2.26853800 -0.63018700

C 5.61821500 0.67759600 -0.43931000

**TS1c**

E= -1690.58450

1 Imaginary frequency = -87.76 cm^-1^

N 4.15020800 4.07992800 0.66075000

C 3.33478400 3.07238300 -0.01867900

C 3.30924400 1.73299700 0.71742700

O 2.53782300 0.77518600 -0.00969900

C 1.94508200 3.66005200 -0.16247200

O 1.34081700 3.70625400 -1.21070500

O 0.47140600 1.63263900 1.12138700

P 1.02470300 0.35392100 0.59478000

N 0.57685900 -0.44084200 -0.78442500

C -0.03704300 -1.75813300 -0.81347400

C 0.33339200 0.36003000 -1.97803200

O 1.51158700 -0.77280100 1.62716500

C 1.05648000 -0.80674700 3.00408800

C 0.58833200 -2.20854600 3.32265100

C 3.80745900 -3.76955400 -1.01920200

N 4.20835700 -3.27693200 -2.23996400

C 3.33566000 -2.70635800 -0.31369200

C 3.99224800 -1.96398100 -2.28539900

N 3.46586500 -1.60311300 -1.12432200

C -5.94378300 -1.85559900 0.88564100

O -1.19587200 -0.58205500 1.39598900

N -2.12330700 -0.03451200 0.65896000

C -3.30905400 -0.55862100 0.74200200

C -4.42875600 -0.02997200 -0.03825600

C -4.26794800 1.09560000 -0.86517900

C -6.57343700 0.99386700 -1.55880500

C -6.74613600 -0.11942500 -0.74133500

C -5.32012900 1.60171100 -1.61578000

H -5.75335300 -1.64145000 1.94027400

H -6.97751100 -2.18887200 0.79085500

H -7.71725800 -0.59991300 -0.68876400

H -7.40245800 1.37984600 -2.13866800

H -5.16280000 2.47089800 -2.24359200

H -3.29387900 1.56522000 -0.90246900

H -3.46250400 -1.40760300 1.40614200

H 1.53053000 4.10521600 0.75575400

H 3.91186000 4.10397500 1.64791700

H 5.12763700 3.81495800 0.60963600

H 3.72008600 2.91567700 -1.02698700

H 2.90771000 1.86649300 1.72390200

H 4.32883800 1.35247700 0.79448000

H 3.16991600 -0.65185300 -0.85800500

H 4.20866500 -1.31701400 -3.11638700

H 2.91538800 -2.63018400 0.67317600

H 4.60627400 -3.81881100 -2.99356600

H 3.89096600 -4.81044300 -0.76679600

H 1.37919400 -2.93461600 3.12549300

H 0.31398400 -2.27633900 4.37737900

H -0.28305800 -2.43433300 2.71069100

H 0.25096400 -0.08882600 3.11926200

H 1.91048400 -0.51487800 3.61558700

H 0.39348700 -2.31157000 -1.65434900

H 0.17228600 -2.28645000 0.11117300

H -1.11925100 -1.68765300 -0.93917400

H 0.73363500 -0.16521200 -2.84994100

H -0.73938000 0.52005900 -2.12451000

H 0.82986300 1.32595200 -1.90016500

H -5.29297400 -2.68725100 0.60438100

C -5.69970300 -0.64333900 0.01940800

**INc**

E= -1690.59526

N 3.97600900 3.77278000 1.37781000

C 3.29313900 2.90805300 0.41016800

C 3.02499300 1.49814400 0.93946200

O 2.34969000 0.72652900 -0.02880000

C 2.00375400 3.60740600 0.03423000

O 1.66938300 3.83761800 -1.10770200

O 0.18941800 1.63693900 0.77791400

P 0.56562100 0.23025100 0.38773500

N 0.55963100 -0.39507300 -1.16966900

C -0.08356400 -1.64486500 -1.53114800

C 0.96697200 0.40610000 -2.31309300

O 1.28153500 -0.78876600 1.46360900

C 0.74416500 -1.02131000 2.77915600

C 0.27746100 -2.45699900 2.91039200

C 4.05882300 -3.50417900 -0.91847400

N 4.91570000 -2.73098300 -1.66602500

C 3.20623000 -2.63502500 -0.30602400

C 4.58861800 -1.44368300 -1.50867300

N 3.55719800 -1.36194700 -0.68630300

C -5.71119600 -1.14814600 1.75262900

O -0.96905000 -0.57088800 0.78841700

N -2.01714500 -0.04341500 0.08916000

C -3.13766900 -0.51077900 0.48378400

C -4.39599700 -0.11110100 -0.16911800

C -4.37111400 0.56986500 -1.39285900

C -6.77376000 0.65153100 -1.42791700

C -6.80391400 -0.02840400 -0.21441000

C -5.54885800 0.95332600 -2.01961500

H -5.17527900 -0.61377900 2.53962900

H -6.75076000 -1.24977300 2.06269100

H -7.75669800 -0.26156900 0.24645300

H -7.69975300 0.94148000 -1.90811800

H -5.51214200 1.47803700 -2.96580500

H -3.41224300 0.78875700 -1.84438000

H -3.16332700 -1.21619200 1.31120200

H 1.40339100 3.96224600 0.88631200

H 3.51127400 3.69678800 2.27828400

H 4.91938100 3.42916000 1.52170500

H 3.89426600 2.84242600 -0.49818900

H 2.44085500 1.56068800 1.86158500

H 3.98748800 1.03022400 1.17877100

H 3.00713200 -0.38947400 -0.37624000

H 5.09099300 -0.61777500 -1.98067000

H 2.38463900 -2.80780300 0.36533400

H 5.67159000 -3.07146600 -2.24088600

H 4.13287000 -4.57594500 -0.89040100

H 1.09902100 -3.14343100 2.69722200

H -0.07267300 -2.64745100 3.92701000

H -0.53659400 -2.64694900 2.21203900

H -0.06675100 -0.32372700 2.97976500

H 1.55896500 -0.81431800 3.47494500

H 0.53117600 -2.14158200 -2.28802200

H -0.16026000 -2.30045300 -0.66738900

H -1.08725600 -1.48808500 -1.94204200

H 1.81944000 -0.05121200 -2.82927100

H 0.13501200 0.47770800 -3.02270000

H 1.24832400 1.40670300 -1.99876200

H -5.28299400 -2.15107500 1.68610000

C -5.62991000 -0.41941800 0.43242600

**TS2c**

E= -1690.59511

1 Imaginary frequency = -117.26 cm^-1^

N 4.02724500 3.74875200 1.35468300

C 3.31911300 2.89012200 0.39973000

C 3.03509600 1.48735900 0.93905800

O 2.34949500 0.71718700 -0.02366800

C 2.03395200 3.60634100 0.04188300

O 1.69055000 3.85124300 -1.09401200

O 0.19621100 1.63943500 0.78812300

P 0.55680000 0.22756500 0.40402600

N 0.54817500 -0.40442600 -1.15041300

C -0.09739300 -1.65623900 -1.50062200

C 0.94429100 0.39240400 -2.30043300

O 1.27490600 -0.79022600 1.47700100

C 0.73578300 -1.03784300 2.78927800

C 0.26923500 -2.47505600 2.90271100

C 4.06462700 -3.49779300 -0.93950400

N 4.91289500 -2.71967100 -1.69158300

C 3.21324400 -2.63261700 -0.31922700

C 4.58065300 -1.43383600 -1.52848200

N 3.55510900 -1.35692700 -0.69885200

C -5.72894700 -1.12441200 1.74837900

O -0.98065900 -0.55985200 0.80956300

N -2.02472500 -0.03071500 0.10411000

C -3.14699600 -0.50220900 0.48878400

C -4.40099300 -0.10242100 -0.17240900

C -4.36725000 0.57078600 -1.40019700

C -6.76924200 0.66029200 -1.44797200

C -6.80812700 -0.01271800 -0.23089600

C -5.54045000 0.95430900 -2.03536800

H -5.18023800 -0.59706200 2.53114000

H -6.76912600 -1.20502000 2.06260000

H -7.76410500 -0.23966900 0.22650600

H -7.69181500 0.95016900 -1.93481500

H -5.49747200 1.47243000 -2.98491800

H -3.40516600 0.78302300 -1.84805300

H -3.17719300 -1.21137000 1.31295100

H 1.44397900 3.95636200 0.90328000

H 3.55652800 3.70275900 2.25425600

H 4.95761900 3.37507500 1.50941600

H 3.90607700 2.81224400 -0.51682700

H 2.45044800 1.56357900 1.85973300

H 3.99124200 1.00870300 1.18227700

H 2.99657100 -0.37535700 -0.37656100

H 5.07701000 -0.60532300 -2.00241900

H 2.39694300 -2.81128500 0.35722200

H 5.66528600 -3.05535100 -2.27375600

H 4.14287600 -4.56934100 -0.91443700

H 1.09207100 -3.15895800 2.68645400

H -0.08636200 -2.67640700 3.91531100

H -0.54120700 -2.65758600 2.19817000

H -0.07557100 -0.34305000 2.99716100

H 1.55008600 -0.83874400 3.48774500

H 0.51087600 -2.15511200 -2.26120200

H -0.16443300 -2.30875100 -0.63353000

H -1.10523600 -1.50253800 -1.90239500

H 1.78968300 -0.06898300 -2.82442800

H 0.10491800 0.46521900 -3.00120400

H 1.23229900 1.39271700 -1.99197100

H -5.32018400 -2.13564200 1.68461400

C -5.63881800 -0.40340100 0.42455400

**C2c**

E= -1690.62399

N -3.73921500 3.52580000 -2.26821600

C -3.63547900 3.00395000 -0.90203700

C -3.47760600 1.48592700 -0.83625400

O -3.31132000 1.11159300 0.51380300

C -2.41917000 3.64527600 -0.27351100

O -2.43977100 4.30284000 0.74378100

O -0.01381500 1.46582600 -0.81269200

P -0.01239500 0.23591500 0.01423000

N -0.07403600 0.47006100 1.63683100

C -0.29652100 -0.68644000 2.50035000

C -0.56343700 1.73940500 2.17322600

O -1.11889800 -0.86627200 -0.31707500

C -1.33553300 -1.32074500 -1.68002900

C -0.71580300 -2.68684700 -1.88446500

C -4.30830600 -3.83131400 0.72262500

N -5.52233300 -3.26448300 0.42664700

C -3.42831200 -2.78592200 0.78911600

C -5.34218800 -1.92925200 0.32741800

N -4.08462500 -1.60272800 0.54061100

C 5.78894000 -2.24749600 -1.30825400

O 1.30927200 -0.72074800 -0.23389400

N 2.49547300 -0.01067200 0.03171900

C 3.50546800 -0.73097300 -0.25533200

C 4.87163700 -0.23074900 -0.04032400

C 5.08101200 0.96093200 0.66481500

C 7.45477000 0.72722100 0.38119500

C 7.24960600 -0.45800900 -0.31719800

C 6.36501300 1.44171800 0.87533800

H 5.17985700 -2.10282500 -2.20276000

H 6.75874100 -2.63113300 -1.62220300

H 8.10025400 -1.00939600 -0.69945200

H 8.46208500 1.09018700 0.54090200

H 6.51609600 2.36271700 1.42300400

H 4.22430600 1.49876800 1.04873000

H 3.35580500 -1.72722900 -0.66144900

H -1.47672600 3.46760300 -0.81717200

H -2.99789900 3.12411800 -2.83536900

H -4.60891500 3.20269600 -2.67849300

H -4.50974500 3.31286400 -0.32823300

H -2.60484800 1.20536900 -1.43858500

H -4.36531600 1.01980200 -1.28178800

H -3.43399500 0.13560700 0.56233900

H -6.14514200 -1.24689300 0.10198600

H -2.37184400 -2.80651000 0.99680400

H -6.39434000 -3.75380500 0.30420200

H -4.18712900 -4.89169100 0.85554600

H -1.14487100 -3.40422000 -1.18444600

H -0.91926600 -3.03027100 -2.90027400

H 0.36256200 -2.64585800 -1.73605800

H -0.92713200 -0.58214100 -2.37076900

H -2.41675700 -1.35214800 -1.79712900

H -1.36478000 -0.89160100 2.62273200

H 0.18886300 -1.56649100 2.08089200

H 0.14057000 -0.48354800 3.47883100

H -1.64303300 1.71282700 2.32870400

H -0.05495200 1.93136000 3.11962200

H -0.33361800 2.54110600 1.47664500

H 5.30791000 -3.01574700 -0.69880600

C 5.96567600 -0.95901500 -0.54100300

**3-hydroxy-2-pyridinealdoxime**

E= -490.25176

O -3.67654000 -0.16184200 -0.00013900

N -2.47114400 -0.61289500 -0.00217900

C -1.51367800 0.28760800 0.00112900

C -0.11501500 -0.08804200 0.00122700

C 0.87581700 0.93103900 0.00010900

C 2.57671000 -0.75366400 -0.00064700

C 1.55577700 -1.69034600 0.00020600

N 0.25545500 -1.38205700 0.00124000

C 2.21544000 0.59624400 -0.00077700

H 1.79104000 -2.75018800 0.00029300

H 3.61474500 -1.05656500 -0.00131000

H 2.96843300 1.37728000 -0.00159900

H -1.77922400 1.34113000 0.00428400

O 0.44322500 2.22920300 -0.00053500

H 1.21103700 2.81707500 0.00281400

**C1d**

E= -1742.48954

N -5.89040800 -2.73711500 -0.29699800

C -4.74850700 -1.92163800 -0.70172800

C -4.17172100 -1.10533700 0.45533200

O -3.09322800 -0.26471900 -0.00668000

C -3.70700700 -2.86171300 -1.27590400

O -3.13283800 -2.66545900 -2.32232100

O -1.43031800 -2.16183400 0.49243800

P -1.54970900 -0.68947300 0.32024900

N -0.78933200 0.15334400 -0.87205000

C 0.63563300 0.46930900 -0.72885700

C -1.22556500 0.03630200 -2.26310200

O -1.16013600 0.15991600 1.61548000

C -0.94641800 -0.49740300 2.89965500

C -0.31174600 0.52337200 3.81428300

C -1.64599900 4.41871300 -0.06159800

N -2.71882200 4.66876600 -0.88760600

C -1.70418900 3.09811400 0.25640200

C -3.41291100 3.54980600 -1.07219000

N -2.81072200 2.58882000 -0.38469800

O 1.84214200 -0.87529000 1.88060000

N 2.90752700 -1.20693700 1.22186900

C 3.66038000 -0.21870900 0.81129300

C 4.88002800 -0.44922300 0.05496700

C 5.63616500 0.66695200 -0.38889400

C 7.20797000 -0.83309500 -1.39252400

C 6.41747900 -1.87158600 -0.92481900

N 5.29274300 -1.69707500 -0.22577700

C 6.79796700 0.47146500 -1.11231700

H 6.70009500 -2.90111400 -1.12031300

H 8.11100800 -1.02764700 -1.95439400

H 7.37464100 1.32465900 -1.45343400

H 3.36955500 0.80304600 1.04056600

H -3.52100200 -3.77164000 -0.68181600

H -5.67306900 -3.24524700 0.55475400

H -6.68181400 -2.14010600 -0.08541300

H -5.04972500 -1.24390900 -1.50130500

H -3.81763200 -1.76668800 1.24713500

H -4.93871000 -0.44410200 0.85419700

H -3.10824400 1.61230200 -0.34239100

H -4.29953700 3.44575300 -1.67144600

H -1.07522500 2.47895800 0.87151000

H -2.95313000 5.56171800 -1.29757400

H -0.95097900 5.18737500 0.22150200

H -0.93804500 1.41257800 3.90041300

H -0.18210000 0.09186000 4.80830000

H 0.66567200 0.79110900 3.41616100

H -0.28196200 -1.33981700 2.73906500

H -1.91497800 -0.83318900 3.27144500

H 0.80411100 1.49132400 -1.07722200

H 0.95017400 0.36440900 0.30910200

H 1.23862000 -0.21219700 -1.33575800

H -1.06484100 0.99732500 -2.75612400

H -0.65160300 -0.73022700 -2.79176900

H -2.28003600 -0.21999200 -2.31603400

O 5.16628400 1.91210200 -0.07546900

H 5.77286200 2.57723200 -0.42846500

**TS1d**

E= -1742.48543

1 Imaginary frequency = -88.40 cm^-1^

N 3.96317800 4.19050000 0.72912400

C 3.20451200 3.15322300 0.02863100

C 3.21277400 1.81155300 0.76119300

O 2.49874900 0.82764400 0.01154000

C 1.79639500 3.68582700 -0.14594800

O 1.21518000 3.71463100 -1.20766100

O 0.37129400 1.58655600 1.09940900

P 0.99170500 0.33535400 0.58063200

N 0.61658200 -0.47060900 -0.81463600

C 0.06840700 -1.81548700 -0.86639800

C 0.33944600 0.33465700 -1.99875600

O 1.51358600 -0.76928200 1.62182300

C 1.02012700 -0.83722400 2.98414100

C 0.60285000 -2.26044200 3.27733600

C 3.99869600 -3.65477500 -0.94313800

N 4.40865300 -3.15161700 -2.15660000

C 3.46076900 -2.61009800 -0.25779600

C 4.13439800 -1.85011500 -2.21695500

N 3.56191600 -1.50675800 -1.07245700

O -1.19218400 -0.67062600 1.32298900

N -2.08742700 -0.18522800 0.51467300

C -3.31455100 -0.56182400 0.73367500

C -4.41094300 -0.10893000 -0.11362500

C -5.73346100 -0.51000000 0.19936400

C -6.51916800 0.71483600 -1.70005400

C -5.19913900 1.06748000 -1.93925600

N -4.17476000 0.67217900 -1.17974500

C -6.78630500 -0.09734300 -0.59830500

H -4.94825000 1.69768100 -2.78613500

H -7.31419100 1.05830700 -2.34736300

H -7.79936800 -0.40469600 -0.36224500

H -3.53229000 -1.22850600 1.56344800

H 1.34247100 4.10737900 0.76495900

H 3.69891400 4.20398800 1.70991900

H 4.95094800 3.96270500 0.70321300

H 3.61959700 3.01480200 -0.97042600

H 2.77957600 1.92623900 1.75671300

H 4.24425400 1.47112900 0.86485700

H 3.21397200 -0.56892900 -0.82031100

H 4.34273900 -1.19903700 -3.04677700

H 3.01147600 -2.54672900 0.71709500

H 4.85023600 -3.67941200 -2.89582200

H 4.12238900 -4.68926400 -0.68126000

H 1.42904400 -2.95060000 3.09687900

H 0.30161000 -2.34986700 4.32302600

H -0.24030200 -2.51900400 2.63927600

H 0.18234300 -0.15435300 3.08396200

H 1.84301800 -0.51619800 3.62306600

H 0.55007400 -2.34535300 -1.69443700

H 0.27370000 -2.33762900 0.06296000

H -1.01120700 -1.79455600 -1.02638300

H 0.75100300 -0.16994600 -2.87731000

H -0.73833400 0.46464600 -2.13434900

H 0.80929300 1.31333100 -1.91332500

O -5.90960300 -1.30180500 1.29833400

H -6.85030500 -1.50067200 1.39865400

**INd**

E= -1742.49450

N 4.05352600 3.74046400 1.33221900

C 3.34045300 2.88588000 0.37724200

C 3.06004400 1.48075000 0.91323500

O 2.36352600 0.71490600 -0.04439000

C 2.05747300 3.60741500 0.02170200

O 1.70828400 3.84244500 -1.11486900

O 0.23948700 1.66976000 0.79451200

P 0.59366000 0.26373400 0.37952200

N 0.54744700 -0.35329100 -1.18175100

C -0.13257500 -1.58281000 -1.54688100

C 0.99392700 0.42818700 -2.32469200

O 1.28219600 -0.78471800 1.44876800

C 0.75135000 -0.98696000 2.77157200

C 0.24891400 -2.40839500 2.92478900

C 3.94275000 -3.59514600 -0.90149700

N 4.82028200 -2.85525300 -1.65907200

C 3.11819300 -2.69611400 -0.29453000

C 4.53455200 -1.55773200 -1.51489000

N 3.50832700 -1.43894700 -0.68971600

O -0.96444700 -0.51354400 0.78471000

N -2.00989800 0.04785400 0.11610500

C -3.12444300 -0.46172200 0.47721300

C -4.39494200 -0.02151500 -0.12323200

C -5.59128100 -0.62106000 0.31016800

C -6.77259500 0.77585100 -1.22689600

C -5.54300300 1.31569700 -1.59650900

N -4.38671900 0.93078100 -1.06277600

C -6.79560700 -0.21244900 -0.25355000

H -5.49027700 2.08948300 -2.35377700

H -7.68718000 1.12055300 -1.68929700

H -7.72743400 -0.66319100 0.06745200

H -3.15387200 -1.23539100 1.23894500

H 1.47940400 3.97638000 0.88315700

H 3.59971700 3.67608800 2.23919400

H 4.99176800 3.37842200 1.46444700

H 3.92544900 2.80935400 -0.54079400

H 2.49039100 1.55360700 1.84358400

H 4.02002600 1.00151800 1.13960500

H 3.00506700 -0.46752600 -0.39291800

H 5.05947400 -0.75123700 -1.99568500

H 2.29349000 -2.83481800 0.38077500

H 5.56416000 -3.22414700 -2.23224200

H 3.98441100 -4.66828200 -0.86330700

H 1.05048800 -3.11869700 2.71355200

H -0.09749100 -2.57746600 3.94644500

H -0.57558800 -2.58501700 2.23521500

H -0.04002200 -0.26652800 2.96976800

H 1.57653300 -0.79208600 3.45885700

H 0.46465300 -2.09279200 -2.30897100

H -0.22489300 -2.24073600 -0.68668600

H -1.13258300 -1.39542400 -1.95323600

H 1.84964300 -0.04680600 -2.81939800

H 0.17784700 0.50404200 -3.05148400

H 1.28327700 1.42822100 -2.01624000

O -5.51427500 -1.58126400 1.26831200

H -6.39955000 -1.91473600 1.46934000

**TS2d**

E= -1742.49394

1 Imaginary frequency = -132.59 cm^-1^

N 4.11284100 3.68285800 1.35471100

C 3.39527500 2.84370600 0.38995300

C 3.07731000 1.44476900 0.92084200

O 2.37137100 0.69858100 -0.04611400

C 2.12886400 3.58764200 0.02180400

O 1.79199400 3.82636500 -1.11745200

O 0.24363900 1.66753200 0.78793400

P 0.56741100 0.25277900 0.38466400

N 0.52992400 -0.36013900 -1.17499600

C -0.14396400 -1.59436200 -1.53639900

C 0.96937400 0.42595900 -2.31733900

O 1.26173600 -0.79239200 1.44784300

C 0.73922400 -0.98998100 2.77570600

C 0.22643700 -2.40723000 2.93157800

C 3.95778400 -3.57597400 -0.92261000

N 4.83256700 -2.82961800 -1.67642400

C 3.13111300 -2.68083400 -0.31156100

C 4.53997400 -1.53310400 -1.52342900

N 3.51412200 -1.41878600 -0.69824400

O -0.98690700 -0.50658600 0.79344200

N -2.02939700 0.05756000 0.11727400

C -3.14569100 -0.44587800 0.47999700

C -4.41398200 -0.00463800 -0.12458600

C -5.61200900 -0.60154900 0.30698200

C -6.78654900 0.79426200 -1.23609300

C -5.55496400 1.33158300 -1.60357900

N -4.40100500 0.94587000 -1.06584400

C -6.81422100 -0.19192200 -0.26100100

H -5.49900000 2.10378000 -2.36217600

H -7.69923600 1.13960600 -1.70173300

H -7.74773200 -0.64037200 0.05825200

H -3.17863100 -1.21531600 1.24579700

H 1.54761400 3.96386200 0.87833200

H 3.64401400 3.63015700 2.25474700

H 5.04059800 3.29985900 1.50141400

H 3.98942400 2.75637400 -0.52120900

H 2.49640800 1.52942300 1.84314100

H 4.02216400 0.94162800 1.15859000

H 2.98545700 -0.41907400 -0.38731200

H 5.06358300 -0.72372600 -2.00117000

H 2.30718700 -2.82880900 0.36301000

H 5.57736000 -3.19264000 -2.25180400

H 4.00288200 -4.64923200 -0.88987700

H 1.02007900 -3.12350200 2.71105800

H -0.11116500 -2.57461100 3.95641400

H -0.60630200 -2.57627200 2.25005700

H -0.04444700 -0.26272300 2.97980500

H 1.57172600 -0.80168500 3.45562400

H 0.45553200 -2.10367500 -2.29688600

H -0.23213700 -2.25008400 -0.67389500

H -1.14506600 -1.41246400 -1.94231700

H 1.81820400 -0.05140500 -2.82090000

H 0.14742100 0.51076900 -3.03640000

H 1.26760000 1.42214700 -2.00482100

O -5.53893100 -1.55987800 1.26691400

H -6.42513300 -1.89159800 1.46683400

**C2d**

E= -1742.52270

N -3.82808000 3.51690700 -2.14060600

C -3.75303100 2.96781700 -0.78320200

C -3.55524000 1.45384900 -0.74508700

O -3.40349400 1.05753100 0.60098400

C -2.57251800 3.62418000 -0.10355500

O -2.64443900 4.28360100 0.91002000

O -0.03927800 1.51341300 -0.85244600

P -0.00151200 0.28453200 -0.02459200

N -0.03446800 0.52521900 1.59880000

C -0.25246700 -0.62844100 2.46794000

C -0.53263700 1.79527300 2.12823400

O -1.10311100 -0.82946700 -0.32971100

C -1.33699400 -1.29160600 -1.68788600

C -0.70540200 -2.65148700 -1.89687100

C -4.13427200 -3.93511200 0.63752700

N -5.35893600 -3.43663200 0.27084500

C -3.33168300 -2.83876600 0.79786200

C -5.26040400 -2.09007900 0.22405500

N -4.04498600 -1.69175000 0.53734300

O 1.32616000 -0.65722500 -0.29479900

N 2.50882800 0.06973400 -0.05913500

C 3.51499700 -0.67532400 -0.28767200

C 4.88462000 -0.16324600 -0.11622700

C 5.95987700 -1.04070900 -0.32358200

C 7.42976900 0.76607700 0.20356700

C 6.30024800 1.56639200 0.38618800

N 5.06105500 1.11666700 0.23155200

C 7.25793500 -0.55948000 -0.15728500

H 6.40624400 2.60731400 0.66721400

H 8.42006800 1.17728100 0.34204700

H 8.10624100 -1.21632700 -0.30780500

H 3.38022400 -1.70305600 -0.60894000

H -1.60511500 3.45475100 -0.60590300

H -3.05868300 3.14732200 -2.69193100

H -4.67550000 3.18051400 -2.58524700

H -4.65178400 3.24290300 -0.23053200

H -2.66331400 1.21048900 -1.33538900

H -4.42037800 0.97177300 -1.21696200

H -3.48612400 0.07651500 0.62584000

H -6.08874000 -1.45284200 -0.03857600

H -2.29216100 -2.80101300 1.07686300

H -6.18667800 -3.97534800 0.07161800

H -3.95315700 -4.98944400 0.74768100

H -1.11585100 -3.36969000 -1.18668600

H -0.92159300 -3.00131300 -2.90786800

H 0.37478000 -2.59814300 -1.76631800

H -0.94846900 -0.55069100 -2.38757000

H -2.41941500 -1.33554600 -1.78709600

H -1.31986800 -0.83271000 2.59872800

H 0.22950100 -1.51000600 2.04755200

H 0.19199300 -0.42283800 3.44240600

H -1.61922800 1.78592700 2.22737400

H -0.07007100 1.96322800 3.10200800

H -0.24716300 2.60280500 1.45909800

O 5.68324100 -2.31906400 -0.67431000

H 6.50360900 -2.81718700 -0.79624500
