# Supplementary material for: In Silico Studies in Probing the Role of Kinetic and Structural Effects of Different Drugs for the Reactivation of Tabun-Inhibited AChE
Source: PLoS One. 2013 Dec 2;8(12):e79591. doi: 10.1371/journal.pone.0079591 (PMC3846473; doi:10.1371/journal.pone.0079591)
Supplement: Table S1 — Binding energies (kcal/mol) calculated with MMFF force field of drug-tabun-conjugated-AChE complexes. (DOC) [file pone.0079591.s008.doc]

**Table S1:** Binding energies (kcal/mol) calculated with MMFF force field of drug-tabun-conjugated-AChE complexes.

|  | Binding Energy | |
| --- | --- | --- |
|  | 100 Å (x, y, z)  grid box | in presence of water at 70Å (x, y, z) grid box |
| Ortho7 | -30.5 | -46.8 |
| 2-PAM | -27.3 | -32.8 |
| DZP | -20.9 | -13.4 |
| 3-hydroxy-2-pyridinealdoxime  **1**  **2** | -10.7  -25.0  -26.0 | -17.6  -21.3  -31.7 |
